# Exploring new pathways in endocrine-resistant breast cancer

**DOI:** 10.37349/etat.2022.00086

**Published:** 2022-06-20

**Authors:** Inês Soares de Pinho, Catarina Abreu, Inês Gomes, Sandra Casimiro, Teresa Raquel Pacheco, Rita Teixeira de Sousa, Luís Costa

**Affiliations:** 1 Oncology Division, Hospital de Santa Maria, Centro Hospitalar Universitário Lisboa Norte, 1649-028 Lisboa, Portugal; 2 Luis Costa Laboratory, Instituto de Medicina Molecular-João Lobo Antunes, Faculdade de Medicina de Lisboa, 1649-028 Lisboa, Portugal; University of Edinburgh, UK

**Keywords:** Breast cancer, endocrine therapy, resistance mechanisms, receptor activator of nuclear factor kappa B ligand/receptor activator of nuclear factor kappa B, nuclear factor kappa B, Notch

## Abstract

The most common breast cancer (BC) subtypes are hormone-dependent, being either estrogen receptor-positive (ER^+^), progesterone receptor-positive (PR^+^), or both, and altogether comprise the luminal subtype. The mainstay of treatment for luminal BC is endocrine therapy (ET), which includes several agents that act either directly targeting ER action or suppressing estrogen production. Over the years, ET has proven efficacy in reducing mortality and improving clinical outcomes in metastatic and nonmetastatic BC. However, the development of ET resistance promotes cancer survival and progression and hinders the use of endocrine agents. Several mechanisms implicated in endocrine resistance have now been extensively studied. Based on the current clinical and pre-clinical data, the present article briefly reviews the well-established pathways of ET resistance and continues by focusing on the three most recently uncovered pathways, which may mediate resistance to ET, namely receptor activator of nuclear factor kappa B ligand (RANKL)/receptor activator of nuclear factor kappa B (RANK), nuclear factor kappa B (NFκB), and Notch. It additionally overviews the evidence underlying the approval of combined therapies to overcome ET resistance in BC, while highlighting the relevance of future studies focusing on putative mediators of ET resistance to uncover new therapeutic options for the disease.

## Introduction

Breast cancer (BC) is the most common solid malignancy in females. Accounting for nearly one in four newly diagnosed cancer cases, BC is currently the leading cause of death in women worldwide [1].

The disease is characterized by remarkable clinical, morphological, and molecular heterogeneity. At the molecular level, BC is mainly classified as hormone receptor-positive (HR^+^), according to the expression of estrogen receptor (ER) and progesterone receptors (PRs), and/or human epidermal growth factor receptor 2-positive (HER2^+^; HER2/ERBB2), if *HER2* oncogene is amplified. Tumors lacking expression of all three receptors are classified as triple-negative BCs (TNBCs). These distinct molecular subtypes have different clinical outcomes and therapeutic options [2].

Approximately 70% of all BCs are ER^+^, with proliferation tightly linked to the ER signaling pathway [3]. The ER is an intracellular receptor activated by estrogen that acts as a transcription factor, regulating the expression of genes related to BC tumorigenesis, proliferation, and survival [4]. ER exists in two isoforms, ERα and ERβ, which can homo- or hetero-dimerize to mediate transcriptional activity. ERα, encoded by the gene estrogen receptor 1 (*ESR1*) on chromosome 6, is ubiquitously expressed in multiple organs, including the mammary gland, acts as a promoter of tumorigenesis, and is the main predictive biomarker of endocrine therapy (ET) efficacy [5, 6].

Previous studies in ERα knockout mice have shown that ERα is essential for the onset of BC development and progression [7]. Consequently, inhibition of ERα through ET has become one of the major strategies for the prevention and treatment of BC.

ERβ, encoded by *ESR2* on chromosome 14, is highly expressed in the prostate and ovaries and appears to have the opposite effect of ERα, restricting estrogen-dependent cell proliferation. In fact, some reports correlated ERβ higher expression with better survival and response to tamoxifen (TAM), irrespective of the ERα status, with lower levels of ERβ potentially contributing to endocrine resistance [8, 9]. This view of ERβ as purely suppressive in BC is defective, with a growing body of evidence reporting elevated ERβ expression as a predictor of poor prognosis and reduced disease-free survival (DFS) in women with ERα^+^ BC who underwent ET [10, 11].

**Figure 1.**
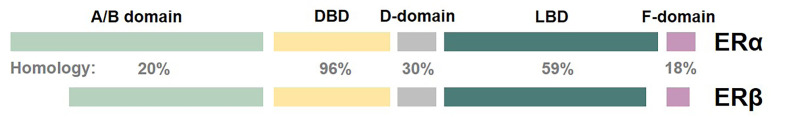
Schematic representation of the structural domains and percentage of homology of ERα and ERβ

Alternative messenger RNA (mRNA) splicing affects the expression of ERα and ERβ, leading to distinctive cellular localization, ligand-binding properties, and post-translational modifications of both receptors. ERα and ERβ have five structural and functional domains, namely, the amino-terminal domain (A/B domain), DNA binding domain (DBD/C-domain), hinge region domain (D-domain), ligand-binding domain (LBD/E-domain), and the carboxyl-terminal domain (F-domain) whose function remains unclear. ERα and ERβ share 96% homology in the DBD region, however, the A/B domain, D-domain, and F-domain are divergent (Figure 1) [12]. At the present moment, the function of ERβ in BC cancer remains a matter of debate and its role in the pathophysiology of estrogen signaling and endocrine resistance is still not fully understood.

For newly diagnosed BC, the pathological determination of positive ER status is an essential criterion for ET treatment. ERα status is determined by immunohistochemistry (IHC) staining on formalin-fixed paraffin-embedded (FFPE) tumor tissue sections, with positivity if ER is detected in ≥ 1% tumor cells, as this predicts responsiveness to ET [13].

Due to the pivotal role of ER signaling in ER^+^ BC, ET constitutes the treatment backbone in this subtype. ET is associated with a mortality reduction of 25–30% in the palliative setting [7] and a nearly 40% reduction in the relative risk of recurrence in the adjuvant setting [8]. Several ET options with different mechanisms of action have been developed over the past decades and are currently available in clinical practice, including inhibitors of ER activity, modulators of ER half-life, and regulators of estrogen availability [3, 14].

**Figure 2.**
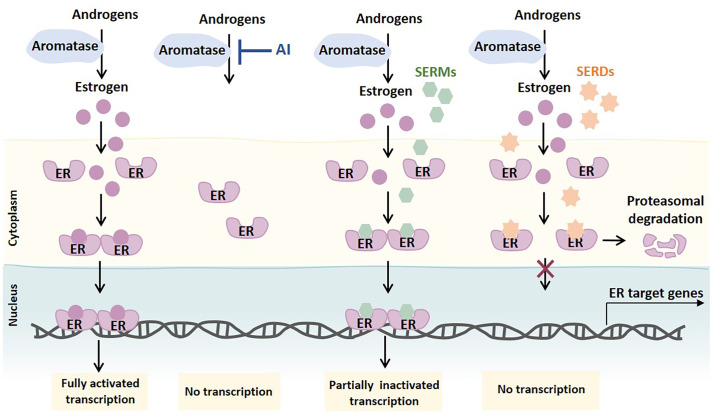
Mechanism of action of ETs primarily used to treat ER^+^ BC. Androgens produced by different tissues are converted into estrogens by aromatase. Upon estrogen binding, ER dimers translocate into the nucleus as transcriptionally active ER complexes. AIs block the synthesis of estrogen by blocking androgen aromatization. SERMs competitively bind to ER and partially impair ER signaling by forming an inactive ER complex. SERDs, considered pure ER antagonists, also competitively bind to ER, but inhibit ER transcription by causing ER complex changes that lead to ER proteasome-dependent degradation

ET agents are mainly divided into three classes: estrogen synthesis inhibitors [e.g., aromatase inhibitors (AIs), as letrozole, anastrozole, and exemestane], selective ER modulators (SERMs; e.g., TAM), and selective ER downregulators [SERDs; e.g., fulvestrant (FULV)]. AIs suppress estrogen production through the inhibition of the enzyme aromatase. Aromatase is responsible for the conversion of androgens produced by different tissues (e.g., ovary, adrenal gland, adipose tissue, breast) into estrogen. By decreasing the levels of estrogen available to bind and activate ER, the ER signaling is impaired. SERMs competitively bind to both ER isoforms, causing conformational changes in the receptor. These changes result in the formation of an inactive ER complex, partially impairing the ER transcriptional activity. SERDs also competitively bind to ER, but the conformational changes induced in the ER complex lead to its degradation, thereby inhibiting its translocation to the nucleus and abolishing ER transcription activity (Figure 2) [3, 14, 15].

Moreover, breast carcinomas that co-express ER and PR tend to have a better response to ET [16, 17]. PR signaling is dependent on ERα, therefore tumors with negative PR status may present altered ERα signaling and therefore an ineffective ET response [18].

However, 40–50% of patients treated with ET are at risk of cancer recurrence or progression due to intrinsic or acquired resistance [19]. Intrinsic or *de novo* resistance—defined as existing prior to treatment start or developing early on the course of treatment—is present in around 50% of patients with metastatic ER^+^ BC [20]. The primary mechanism of intrinsic resistance is the loss of expression of ERα [4]. However, it has been recently shown that patients carrying inactive cytochrome P450 2D6 (CYP2D6) alleles— approximately 8% of Caucasian women—are unable to convert TAM into its active metabolite, endoxifen, being thus less responsive to TAM [21]. Acquired resistance occurs in a significant percentage of patients after initial response during prolonged exposure to ET. The mechanisms responsible for acquired resistance are multiple and include the activation of several signaling pathways. Importantly, some mechanisms involved in intrinsic and acquired resistance overlap.

In this review, the already well-established mechanisms of ET resistance will be revisited, and the evidence underlying three pathways recently reported as putative mediators of ET resistance in BC—receptor activator of nuclear factor kappa B ligand (RANKL)/receptor activator of nuclear factor kappa B (RANK), nuclear factor kappa B (NFκB), and Notch—will be addressed.

## A brief revisit of recognized mechanisms of ET resistance

In recent years, several molecular mechanisms of ET resistance have been the subject of comprehensive reviews, with the complex crosstalk between ER signaling and other signaling pathways responsible for orchestrating the phenomenon now intelligible [12, 22, 23–31]. Since a thorough description of ET resistance mechanisms is beyond the scope of this review, the main mechanisms will be hereinafter briefly summarized (Table 1).

**Table 1.** Mechanisms of endocrine resistance [3]

**Resistance pathway**	**Mechanism**	**Reference(s)**
ER expression and activity loss	Mutations	[32]
	Gene regulation	[33]
	Post-transcriptional modifications (e.g., splice variants, mRNA stability)	[34, 35]
	Post-translational modifications	[36]
Transcriptional machinery of ER	Down-regulation of co-repressors (e.g., NCoR)	[37]
	Over-expression of co-activators (e.g., AIB1)	[38, 39]
	Increased expression of transcriptional factors (e.g., AP-1, SP-1, NFκB)	[40, 41]
Cross-talk between ER and RTKs	EGF/EGFR	[42–44]
	HER2	[44–46]
	IGF1R	[47, 48]
	PI3Ks/Akt	[48–52]
	p44/42 MAPK	[53, 54]
	Stress-induced kinases (JNK, p38 MAPK)	[55]
Cell cycle regulators	Over-expression of positive regulators (e.g., MYC and cyclins E1 and D1)	[56]
	Reduced expression of negative regulators (e.g., p21 and p27)	[57, 58]
	Over-expression of anti-apoptotic molecules (e.g., BCL-XL)	[59]
	Reduced expression of pro-apoptotic molecules (e.g., BCL-2-interacting killer and caspase 9)	[59]

AIB1: amplified in breast 1; Akt: protein kinase B; AP-1: activator protein 1; BCL-2: B-cell lymphoma 2; BCL-XL: B-cell lymphoma-extra large; EGF: epidermal growth factor; EGFR: EGF receptor; IGF1R: insulin growth factor 1 receptor; JNK: c-Jun N-terminal kinase; MAPK: mitogen-activated protein kinase; NCoR: nuclear receptor corepressor; PI3Ks: phosphatidylinositol 3-kinases; SP-1: specificity protein 1

*Note.* Reprinted from “Biological mechanisms and clinical implications of endocrine resistance in breast cancer,” by Giuliano M, Schifp R, Osborne CK, Trivedi MV. Breast. 2011;20 Suppl 3:S42–9 (https://linkinghub.elsevier.com/retrieve/pii/S0960977611702934). CC BY-NC-ND.

### Alterations in ER expression and signaling

ET resistance has been associated with alterations in the ER expression at gene and protein levels or in the expression of *CYP19A1* (or *ARO1*) gene encoding for aromatase. Pre-clinical and clinical evidence supports that ER expression may change during the natural history of disease and under ET exposure, and that, a switch to an ER-negative phenotype results in ET inefficacy. An estimated 20% of ER^+^ BC patients treated with ET lose ER expression over time [3, 12, 60], mainly due to epigenetic and post-transcriptional mechanisms [3, 4, 15, 61]. The first point mutations in the *ESR1* gene, which encodes for ERα, were reported two decades ago [62]. However, it was only more recently that these mutations were found to enable hormone-independent ER transcriptional activity, resulting in constitutive ER activity and ET resistance [4, 60, 61, 63–65]. Such mutations mostly occur in the LBD of *ESR1* [4, 64, 65] and are detected in 20% of recurrent BCs following long-term treatment with AIs or TAM [63]. Although at a much lower frequency than point mutations, *ESR1* amplification and gene fusions have also been reported [63–65].

Next-generation oral SERMs or SERDs (e.g., elacestrant, SAR43985, rintodestrant, ZN-c5, H3B-6545, and ARV-471) are currently in clinical development to target both wild-type and mutant ER as a way to overcome ER mutation-driven resistance to ET [63].

Additionally, ER transcriptional activity can be modulated by different coregulatory proteins and/or transcriptional factors. Coregulatory proteins can be coactivators [e.g., nuclear receptor coactivator 1 (NCoA1), NCoA2, NCoA3] or corepressors [e.g., NCoR1, nuclear receptor subfamily 2 group F member 2 (NR2F2)] and are recruited to bind to ER and respectively enhance or repress the transcriptional activity of specific DNA elements [estrogen response elements (EREs)] located in the promoter regions of different ER target genes. Moreover, transcriptional factors, like AP-1 and SP-1, regulate the transcription of genes that do not contain EREs. Changes in the expression of coregulatory proteins or transcriptional factors critically influence the effectiveness of ET and are also associated with endocrine resistance [3, 6, 12].

Amplification of the *CYP19A1* gene is also found in AI-resistant breast tumors (21.5% of AI-treated and < 2% of primary tumors), leading to increased aromatase activity, estrogen availability, and consequent ER signaling [66]. Although irreversibly resistant to AIs, these tumors remain sensitive to FULV, being strong candidates for treatment with SERDs [27, 63].

Overall, any variation in ER expression or signaling may contribute to endocrine resistance and more aggressive phenotypes. The regulation of ER expression is complex and not totally understood as reviewed by Hua et al. [66], requiring further investigation.

### Regulators of cell cycle and apoptosis

Cell cycle and apoptosis regulators play a key role in the proliferation of normal and BC cells. Therefore, it comes with no surprise that deregulation of these proteins is associated with endocrine resistance.

Several studies have shown that the activity of both positive and negative cell cycle regulators can have an impact on BC sensitivity to ET [3, 67, 68]. Increased expression of positive regulators, like c-MYC and cyclins D1 and E1, can activate cyclin-dependent kinases (CDKs), contributing to aberrant cell cycle progression and resistance to ET. Reduced activity of negative cell cycle regulators, like p21 and p27, and inactivation of retinoblastoma (RB) tumor suppressor also enable aberrant cell cycle progression, contributing to endocrine resistance. Additionally, increased expression of anti-apoptotic molecules like BCL-XL and reduced expression of pro-apoptotic molecules like BCL-2-interacting killer and caspase 9 also play a role in ET resistance [3, 4, 12].

**Figure 3.**
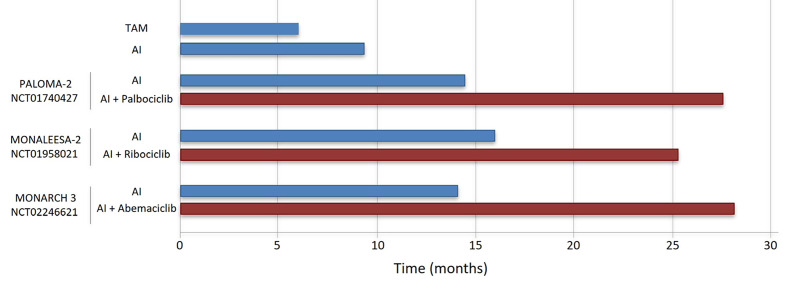
mPFS of ET (blue bars) and combinations with CDK4/6i (red bars) in first line treatment of HR^+^/HER2^–^ advanced BC, as reported in key clinical trials [69, 72, 79, 81]

**Figure 4.**
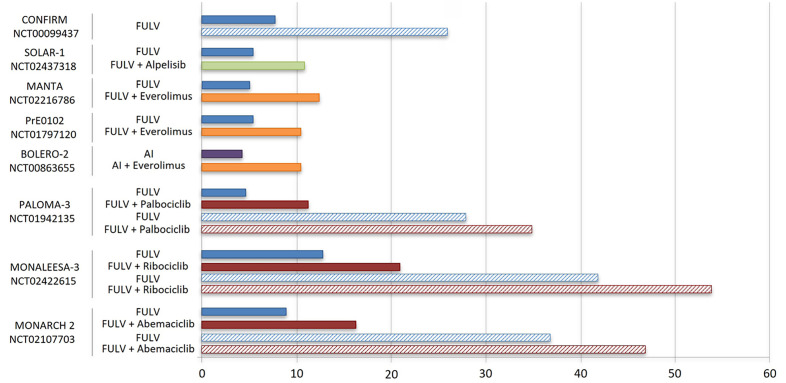
mPFS (solid bars) and mOS (striped bars) of ET and combinations with CDK4/6i, PIK3CA and mTOR inhibitors in second line treatment of HR^+^/HER2^–^ advanced BC, as reported in key clinical trials [71–77, 82–86]

Three CDK4/6 inhibitors (CDK4/6i; palbociclib, ribociclib, abemaciclib) are currently approved in combination with standard ET to treat metastatic ER^+^ BC. Clinical studies showed that combined CDK4/6i and letrozole significantly improve progression-free survival (PFS) compared to letrozole alone in advanced ER^+^ BC resulted in the approval of CDK4/6i in combination with an AI in first line treatment of this disease [69–71]. In addition, the combination of a CDK4/6i and FULV has been approved for use following progression on initial AI monotherapy (Table 2, Figures 3 and 4) [20, 72, 73].

**Table 2.** Summary of clinical trials leading to the approval of new drugs to overcome endocrine resistance in HR^+^/HER2^–^ BC

**Drug**	**Target**	**Study**	**Phase**	**Population**	**Therapy line**	**Treatment arms**	**Outcome**	**Reference(s)**
CDK4/6i								
Palbociclib	CDK4/6	PALOMA-3 NCT01942135	III	HR^+^/HER2^–^ postmenopausal advanced BC	Second or later lines	Palbociclib + FULV *vs*. placebo + FULV	mPFS: 11.2 mo *vs*. 4.6 mo; hazard ratio: 0.46; *P* < 0.0001 mOS: 34.9 mo *vs*. 28 mo; hazard ratio: 0.81; *P* = 0.09	[72, 74]
Ribociclib	CDK4/6	MONALEESA-3 NCT02422615	III	HR^+^/HER2^–^ postmenopausal advanced BC	First or second line	Ribociclib + FULV *vs*. placebo + FULV	mPFS: 21.0 mo *vs*. 13 mo; hazard ratio: 0.59; *P* < 0.001 mOS: 54 mo *vs*. 42 mo; hazard ratio: 0.73; *P* < 0.01	[75, 76]
Abemaciclib	CDK4/6	MONARCH 2 NCT02107703	III	HR^+^/HER2^–^ postmenopausal advanced BC	Second or later lines	Abemaciclib + FULV *vs*. placebo + FULV	mPFS: 16.4 mo *vs*. 9.3 mo; hazard ratio: 0.55; *P* < 0.001 mOS: 46.7 mo *vs*. 37.3 mo; hazard ratio: 0.75; *P* = 0.01	[73, 77]
Palbociclib	CDK4/6	PALOMA-2 NCT01740427	III	HR^+^/HER2^–^ postmenopausal advanced BC	First line	Palbociclib + letrozole *vs*. placebo + letrozole	mPFS: 27.6 mo *vs*. 14.5 mo; hazard ratio 0.56, *P* < 0.001 ORR: 42% *vs*. 35%	[69, 78]
Ribociclib	CDK4/6	MONALEESA-2 NCT01958021	III	HR^+^/HER2^–^ postmenopausal advanced BC	First line	Ribociclib + letrozole *vs*. placebo + letrozole	mPFS: 25.3 mo *vs*. 16 mo; hazard ratio: 0.57; *P* < 0.001 ORR: 43% *vs*. 29%	[79]
	CDK4/6	MONALEESA-7 NCT02278120	III	HR^+^/HER2^–^ premenopausal advanced BC	First line	Ribociclib + letrozole/anastrozole/TAM + goserelin *vs*. placebo + letrozole/anastrozole/TAM + goserelin	mPFS: 24 mo *vs*. 13 mo; hazard ratio: 0.55, *P* < 0.0001 mOS: n.r. *vs*. 40.7 mo; hazard ratio: 0.71; *P* = 0.00973	[70, 80]
Abemaciclib	CDK4/6	MONARCH 3 NCT02246621	III	HR^+^/HER2^–^ postmenopausal advanced BC	First line	Abemaciclib + letrozole *vs*. placebo + letrozole	mPFS: 28.2 mo *vs*. 14.2 mo; hazard ratio: 0.54; *P* < 0.001 ORR: 59% *vs*. 44%	[81]
PI3K/AKT/mTOR inhibitors								
Everolimus	mTOR1	BOLERO-2 NCT00863655	III	HR^+^/HER2^–^ postmenopausal advanced BC	Second or later lines	Everolimus + exemestane *vs*. placebo + exemestane	mPFS: 10.6 mo *vs*. 4.1 mo; hazard ratio: 0.36, *P* < 0.001 ORR: 7% *vs*. 0.4%	[82]
		MANTA NCT02216786	II	HR^+^/HER2^–^ postmenopausal advanced BC	Second or later lines	Everolimus + FULV *vs*. placebo + FULV	mPFS: 12.3 mo *vs*. 5.4 mo; hazard ratio: 0.63, *P* = 0.01	[83]
		PrE0102 NCT01797120	II	HR^+^/HER2^–^ postmenopausal advanced BC	Second or later lines	Everolimus + FULV *vs*. placebo + FULV	mPFS: 10.3 mo *vs*. 5.1 mo; hazard ratio: 0.61, *P* = 0.02	[84]
Alpelisib	Class I PI3K p110α	SOLAR-1 NCT02437318	III	HR^+^/HER2^–^ postmenopausal advanced BC	First or second line	Alpelisib + FULV *vs*. placebo + FULV	mPFS: 11.0 mo *vs*. 5.7 mo; hazard ratio: 0.65, *P* < 0.001 ORR: 26.6% *vs*. 12.8%; PIK3CA- mutant subset mOS: 39.3 mo *vs*. 31.4 mo; hazard ratio: 0.86, *P* = 0.15	[71, 85]

mo: months; mOS: median overall survival; mPFS: median PFS; mTOR: mammalian target of rapamycin; n.r.: not reached; ORR: overall response rate; PIK3CA: phosphatidylinositol-4,5-bisphosphate 3-kinase catalytic subunit alpha

The activity of cell cycle and apoptosis regulators can be modulated by tyrosine kinase receptors (TKRs) and transcription factor signaling pathways, with evidence of crosstalk between ER signaling, TKR pathways, cell cycle regulators, and apoptotic molecules towards ET escape.

### TKRs and alternative pathways

Comprehensive evidence suggests that endocrine resistance can be driven by increased expression and activity of TKRs and subsequent activation of downstream signaling pathways, such as PI3K/AKT/mTOR and MAPK. Upon aberrant activation of TKRs (most commonly by amplification or mutation), downstream intracellular signal transduction pathways are activated, inducing ER phosphorylation and activation in the absence of estradiol [3, 4, 60, 63].

TKRs are a family of cell membrane receptors with a tyrosine kinase intracellular domain that phosphorylates tyrosine residues of target proteins. They include HER2, EGFR, FGFRs, and IGFR. *HER2*/*ERBB2* amplification has long been known to reduce sensitivity to anti-estrogens in ER^+^/HER2^+^ tumors, with the current standard of care for this BC subtype being a combination of ET and HER2 inhibitors. Treatment of HER2-amplified BC will not be explored, as it is out of the scope of this review but has been recently reviewed by Chow et al. [87]. More recently, HER2-activating mutations have been associated with both intrinsic and acquired ET resistance [88, 89]. Somatic mutations in HER2 are observed in approximately 5% of endocrine-resistant, non-HER2-amplified BCs [90]. Tumors with an incidence of HER2 mutations as high as 10% are further enriched in lobular histology [91]. Combined blockade of HER2 mutations and ER results in synergistic antitumor activity *in vitro* and *in vivo* [88, 89], and the combination of neratinib (a tyrosine kinase inhibitor of HER2, EGFR, HER4, and HER3 heterodimers) and FULV has shown promising activity in metastatic ER^+^/HER2-mutated BC [91].

FGFR1–4 constitute a family of four highly conserved TKRs. Deregulation of FGFR has been reported in a variety of human cancers, including BC [92]. FGFR1 amplification is found in approximately 14% of metastatic BCs and is associated with *de novo* endocrine resistance [93]. *FGFR2* genomic and expression alterations can be found in approximately 6% of BCs [94], with FGFR2 associated with endocrine resistance *in vitro* [95]. Clinical trials combining pan-FGFR inhibitors and ET are ongoing in FGFR1/2-amplified ER^+^/HER2^–^ metastatic BC. FOENIX-MBC2 TAS-120-201 (NCT04024436) is a phase II trial evaluating the combination of FULV with futibatinib (TAS-120). The triple combination of ET, CDK4/6i, and FGFR inhibitor is also being explored in a phase Ib trial (VICC BRE 16126; NCT03238196). *FGFR4* genomic and expression alterations can be found in approximately 7% of BC patients [94]. Recently, *FGFR4* overexpression and hotspot mutations have been associated with metastases and endocrine resistance in lobular metastatic BC [96], and a recent study suggested that FGFR4 promotes the transition from a more differentiated luminal phenotype to a highly proliferative and metastatic, endocrine-resistant, HER2-enriched subtype [97]. The combination of ET with an FGFR4-selective inhibitor seems to be an attractive strategy in tumors exhibiting FGFR4 genomic signatures.

The PI3K/AKT/mTOR pathway is one of the most frequently activated pathways in several types of cancer [98] and the link between PI3K/AKT/mTOR pathway deregulation and endocrine resistance in BC is well established [31, 99]. A number of drugs targeting PI3K/AKT/mTOR are currently being investigated in clinical trials in combination with standard therapies as a strategy to overcome acquired resistance in BC. Somatic mutations in PIK3CA are the most common cause of aberrant PI3K/AKT/mTOR pathway activation, prompting the development of various PI3K inhibitors (PI3Ki) with a different selectivity against the four PI3K catalytic subunit isoforms [100]. The selective alpha isoform inhibitor alpelisib (BYL719) was the first oral PI3Ki developed and was recently approved for the treatment of advanced PIK3CA-mutated ER^+^/HER2^–^ BC progressing on previous ET (Table 2, Figure 4) [85]. AKT is another potential therapeutic target to overcome endocrine resistance, with several AKT inhibitors (AKTi) currently under clinical investigation. The combination of the pan-AKT inhibitor capivasertib (AZD5363) with FULV has been shown to improve PFS in metastatic ER^+^ BC progressing on AIs [101]. This combination may be particularly effective in ER^+^ BC with AKT1 mutations [102, 103]. Notably, it has been recently shown that triple inhibition by FULV, CDK4/6i, and AKTi durably impairs BC cell growth, prevents progression, and reduces metastases of tumor xenografts resistant to the combination of CDK4/6i and FULV or FULV alone [104]. A phase III trial (CAPItello-291; NCT04305496) is currently ongoing to evaluate capivasertib in combination with FULV in metastatic ER^+^ BC following progression on AIs. Furthermore, a phase Ib/III trial is evaluating capivasertib plus palbociclib and FULV in locally advanced, unresectable, or metastatic ER^+^ BC (CAPItello-292; NCT0486266).

Inhibition of mTOR, a downstream target of the PI3K pathway, has also been shown to reduce endocrine resistance. The mTOR complex-1 inhibitor everolimus in combination with exemestane was shown to prolong PFS in advanced ER^+^/HER2^–^ BC after progression on non-steroidal AIs [82], independently of PIK3CA mutational status [105]. Consistently, patients with AI-resistant metastatic BC have also been shown to obtain clinical benefits from everolimus combined with FULV (Table 2, Figure 4) [83, 84].

## Recently uncovered mechanisms of ET resistance

### RANKL-RANK pathway

RANKL and its receptor RANK belong to the tumor necrosis factor (TNF) superfamily and were identified in the late 1990s as key regulators of osteoclastogenesis [106, 107]. The pivotal role of the RANKL-RANK pathway in bone physiology and pathology led to the development of denosumab, a fully human anti-RANKL monoclonal antibody currently approved for the treatment of bone remodeling diseases, including cancer-induced bone metastases [108, 109].

In the last decade, extensive research stemming from the observation of RANK-expressing RANKL-sensitive cancer cell lines from different tumor types, including BC [110–113], clearly corroborated that the RANKL-RANK pathway is a major mediator of breast physiology and carcinogenesis [107, 114–116] as recently reviewed by our study group [117]. In view of this evidence, inhibition of the RANK pathway emerged as a promising strategy in BC prevention and treatment.

The fact that expansion of RANK-positive mammary basal stem cells is mediated by paracrine RANKL signaling [115] and that RANK-positive BC cases are more common among TNBC subtypes [118–123] established the relevance of the RANKL-RANK pathway mainly in this BC subtype. Accordingly, numerous studies have shown that RANK-expressing TNBC is a more aggressive subtype, as RANKL activates a signaling cascade involving NFκB, AKT (PKB), JNK, extracellular signal-regulated kinase (ERK), proto-oncogene tyrosine-protein kinase Src (Src), and MAPK, increasing migration, invasion, stemness, transformation, epithelial-mesenchymal transition (EMT), anchorage-independent growth, and metastatic ability [117]. It was only recently that our own research [124] and that of Benítez et al. [125] disclosed a link between RANK expression and luminal BC phenotype and carcinogenesis, respectively. In a recent study, our group characterized the phenotype of RANK-overexpressing (RANK OE) luminal BC cell lines, showing that RANK OE cells have a staminal and mesenchymal phenotype, with decreased proliferation rate and decreased susceptibility to chemotherapy, but are more invasive *in vivo* [124]. *In silico* analysis of the transcriptome of human breast tumors confirmed the association between *RANK* expression and stem cell and mesenchymal markers in ER^+^/HER2^–^ tumors, leading to the hypothesis that luminal RANK-positive cells may constitute an important reservoir of slow cycling, therapy-resistant cancer cells [124].

Prior studies have shown that multiparous mouse mammary tumor virus (MMTV)-RANK mice, with RANK OE in the mammary gland under MMTV, develop spontaneous breast tumors with long latency and only after multiple pregnancies [126], although tumor latency decreases and tumor incidence increases compared to wild-type after carcinogenic protocols [22, 115]. Genetic or pharmacological inhibition of RANK signaling abrogated carcinogenesis in this model and also delayed tumor onset and decreased tumor and metastases incidence in HER2^+^ or polyomavirus-induced BC, MMTV-Neu, and MMTV-polyomavirus middle T antigen (PyMT) mice [127, 128]. In these models, RANK was focally expressed in non-transformed mammary glands but increased in mammary pre-neoplastic lesions and invasive adenocarcinomas. The long latency of MMTV-RANK carcinogenesis was recently found to be due to RANK-driven senescence, which initially delays tumor onset in oncogene-driven models but promotes stemness, luminal-like tumor growth, and metastases in later stages of tumor progression [125, 128]. In accordance with these findings, transcriptomic analysis of human breast tumors from luminal subtype linked *RANK* expression to senescence [125].

The work of our group has also disclosed that RANK OE cells are more resistant to FULV compared to RANK-low counterparts [124]. This suggests that the RANK pathway may be implicated in intrinsic ET resistance. Interestingly, continuous pathway activation by prolonged RANKL exposure led to a decrease in proliferation and down-regulated ER and PR, with an increase in FULV resistance. This suggests that RANK signaling may also be associated with the induction of acquired resistance to ET due to ER loss. Activation of the RANK signaling pathway leads to an increase in noncanonical NFκB signaling, which plays a key role in the above-mentioned regulation of proliferation of mammary epithelial cells—through the RANKL–RANK–NFκB inhibitor (IκB) kinase α (KKα; IKKα)–IκBα–p50/p65–cyclin D1 axis [129]. Unlike the canonical NFκB pathway that responds rapidly to signals from different receptors, the noncanonical NFκB pathway specifically responds to a small group of receptors, namely lymphotoxin-β receptor (LTβR), B-cell-activating factor receptor (BAFFR) belonging to the TNF family receptor, CD40, and RANK [130].

The RANK–NFκB axis has been associated with increased resistance to the anti-HER2 therapy lapatinib in RANK-positive HER2^+^ BC cells [131]. Moreover, although several signaling pathways crosstalk due to activation of different receptors, lapatinib was not able to counteract RANK-induced NFκB activation in this study. Conversely, it was previously reported that NFκB inhibitors abrogate RANKL-induced EMT, cell migration, and invasion [132].

**Figure 5.**
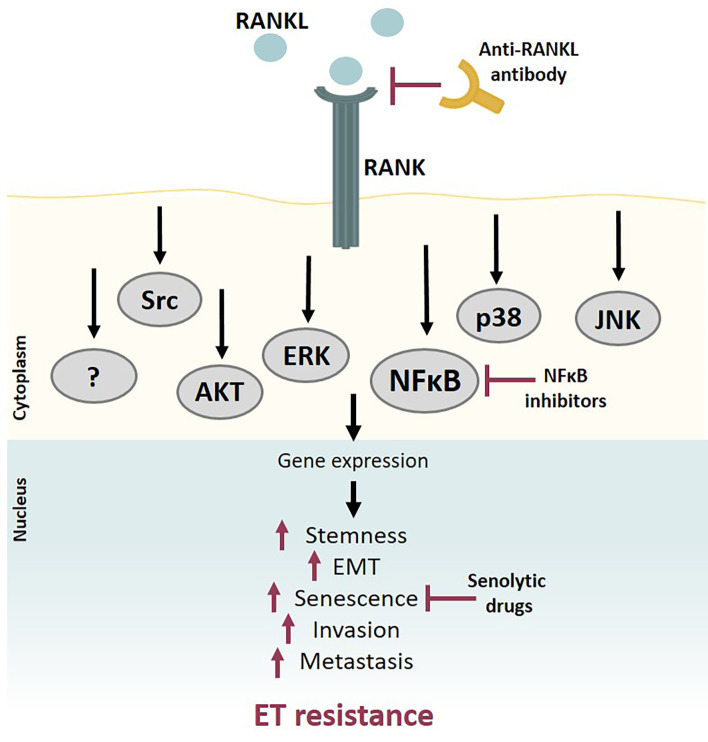
RANK pathway contributes to ET resistance. RANK activation by RANKL activates a signaling cascade that involves several downstream pathways, such as NFκB, AKT, ERK, and MAPK. RANK OE breast tumors are more aggressive, presenting a staminal and mesenchymal phenotype, with increased invasion and metastatic ability. RANKL-RANK pathway activation induces ET resistance through NFκB activation and/or other mechanisms yet to be clarified. Inhibition of RANK pathway signaling, either by blocking RANKL, inhibiting downstream mediators like NFκB, or using senolytic drugs to overcome RANK-induced senescence, may contribute to avoiding or circumventing ET resistance

It is widely accepted and clearly demonstrated that multiple mechanisms are involved in the crosstalk between NFκB and ER. Accordingly, there is compelling evidence of a role for the NFκB pathway in ET resistance, indicating that RANK-mediated NFκB signaling may contribute to ET resistance. This suggests that it may be of particular interest to further study the mechanism of RANK-mediated ET resistance and investigate the efficacy of RANK pathway inhibition—either at the RANKL level or via inhibition of downstream mediators like NFκB—as a mechanism to overcome it (Figure 5).

### NFκB pathway

The NFκB family comprises five inducible transcription factors p65(RelA), RelB, cRel, NFκB1 or p50, and NFκB2 or p52, all of which play critical roles in cell proliferation and survival and inflammatory and immune responses [133–136], being crucial for normal organ development, including of the mammary gland [137].

A growing body of evidence indicates abnormal activation of the NFκB pathway in multiple malignancies, suggesting a putative role for NFκB in tumorigenesis [134, 138–141] and chemotherapy resistance [142].

Members of the NFκB family have a conserved N-terminal Rel homology domain (RHD) [143] that is responsible for dimerization, nuclear translocation, DNA binding, and interaction with IκB. This family of molecules includes multiple proteins, among which IκBα, IκBβ, and IκBε, are the most important NFκB regulators. NFκB molecules exist either as homo or heterodimers, but the most abundant intracellular form is the p50(NFκB1)/p65(RelA) heterodimer. On unstimulated cells, NFκB homo or heterodimers are hijacked in the cell cytoplasm, binding to their IκB inhibitor [143]. Activation of the NFκB cascade occurs either through canonical or noncanonical pathways [144, 145].

The canonical pathway is activated following stimulation by inflammatory cytokines [interleukin-1 (IL-1), lipopolysaccharide (LPS), TNFα, T- and B-cell mitogens], growth factors, viral proteins, and extracellular physical and chemical stress [146, 147]. These ligands activate their receptors and initiate a downstream cascade that ultimately leads to phosphorylation and subsequent ubiquitination of IκBα by a trimeric IKK complex [IKKα, IKKβ, and IKKγ or NFκB essential modulator (NEMO)], enabling nuclear translocation of the p50/p65 heterodimer. Upon translocation, the complex interacts with κB sites to accelerate the transcription of target genes [144, 145, 148–150].

Conversely, in the noncanonical pathway, NFκB-inducing kinase (NIK) phosphorylates and activates IKKα in response to signals from a small group of receptors, including the RANK [151]. Activated KKα then phosphorylates p100, resulting in p52 liberation and promoting p52/RelB nuclear translocation [152, 153].

Contrarily to p50/RelA activation in the canonical pathway, the role of p52/RelB in therapy resistance remains poorly understood. Nevertheless, RelB expression was found to be high in ER^+^ BC cells [154], and inhibition of p52/RelB was shown to be able to reverse ER expression [155].

Regarding ET resistance, comprehensive evidence shows that NFκB regulates a series of genes relevant to endocrine resistance [156–158]. In fact, there seems to be a reciprocal interaction between endocrine treatments and the immune system. Inflammation cytokines like IL-1 and TNFα are higher in metastatic BC patients, where they seem to activate the NFκB pathway and lead to endocrine resistance. Antibodies directed against transcription factors upstream of this pathway have restored sensitivity in cell line models of endocrine-resistant BC [4]. There is also supporting evidence that a dysregulated immune response or excessive inflammation in the tumor microenvironment (TME) could be related to endocrine resistance and could promote BC progression and metastasis [159, 160]. Downstream NFκB-regulated proteins, like BCL-2, cyclin D1, and cytokines IL-6 and IL-8, have been shown to be major players in this process [148, 154]. Additionally, previous studies documented that estrogen withdrawal led to the increased transcriptional activity of p65(RelA) and sustained estrogen-independent tumor growth through upregulation of cyclin D1 and BCL-3 [157]. Oida et al. [161] focused on the role of NFκB in hormone dependency in BC and showed that NFκB inhibition enhanced ER expression and promoted recovery of TAM sensitivity in ER-reduced cell sublines. Another study by Nehra et al. [162] reported p65(RelA) inhibition, either by overexpression of mutant IκB or by synergistic restoration of sensitivity to TAM by the small-molecule NFκB inhibitor parthenolide in resistant MCF-7 cell lines, along with decreased BCL-2 expression and induced caspase-dependent apoptotic cell death in resistant cells. Notably, all effects could be reversed by caspase-8 specific inhibition. In TAM-resistant BC cells, Zhou et al. [134] described the increased transcriptional activity of NFκB and AP-1, which was suppressed after treatment with parthenolide or the proteasome inhibitor bortezomib. Additionally, increased p50/NFκB1, p52/NFκB2, and cRel expression were observed in breast tumors compared with normal surrounding tissues [163].

**Figure 6.**
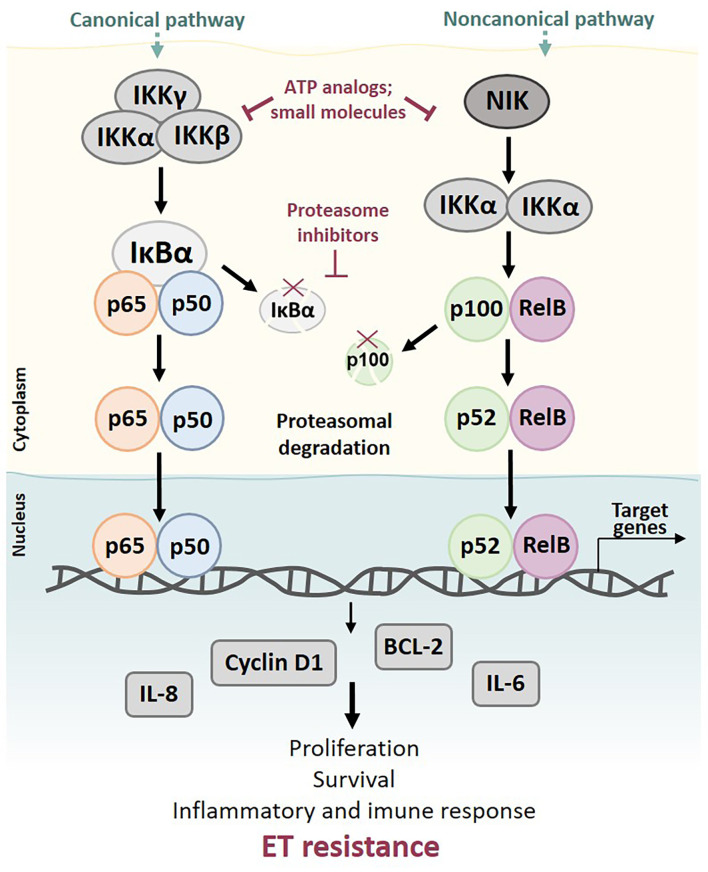
Activation of canonical and noncanonical NFκB pathways may lead to ET resistance. Activation of the canonical pathway can be induced by multiple stimuli, including inflammatory cytokines and growth factors, ultimately leading to phosphorylation and subsequent ubiquitination of IκBα by a trimeric IKK complex (IKKα, IKKβ, and IKKγ), allowing the nuclear translocation of p50/p65 heterodimer. Upon translocation, the complex accelerates the transcription of target genes. The noncanonical pathway is activated by TNF cytokine family members and results in phosphorylation of NIK and activation of IKKα. Activated IKKα then phosphorylates p100, resulting in the liberation of p52 and promoting p52/RelB nuclear translocation. Both canonical and noncanonical pathways play important roles in cell proliferation and survival and inflammatory and immune response. Deregulation of NFκB signaling can lead to drug resistance and expression of downstream NFκB-regulated proteins, like BCL-2, cyclin D1, and cytokines IL-6 and IL-8 described as contributors to ET resistance

Owing to the possible putative role of NFκB in BC, targeting the NFκB pathway might be a successful therapeutic strategy. Several inhibitors have been reported to date, still in early developmental stages (Figure 6).

Most of these inhibitors prevent proteasomal degradation of IκB proteins, resulting in NFκB hijacking in the cytoplasm. To accomplish this, the drug has either to inhibit IκB function (e.g., proteasome inhibitors), phosphorylation of IκB proteins (e.g., parthenolide), or IKKα and IKKβ. Selective IKKβ inhibitors in particular have shown exciting results in preclinical models, but also important toxicities, precluding their use in clinical practice [164]. Another drug class that can block NFκB activity is the class of nuclear translocation inhibitors. Regardless of the mechanism, the use of NFκB inhibitors in BC endocrine resistance may help elucidate target drivers of this phenomenon and contribute to restoring ET sensitivity.

### Notch pathway

It is acknowledged that BC subtypes have different clinical outcomes due to different biological and cellular mechanisms involved in tumor aggressiveness, metastases formation, and treatment response [165]. Increased Notch activity and/or Notch deregulation lead to the transformation of normal breast cells into cancer cells, with differential Notch ligand activation potentially associated with oncogenesis and progression of particular BC subtypes and poor clinical outcomes (Table 3) [166, 167].

**Table 3.** Alterations in Notch pathway according to BC subtype [165]

**Subtype**	**BC subtype**			
	**HER2^+^**	**Basal-like**	**Luminal A**	**Luminal B**
Notch activation (*mRNA*, protein )	- High *Notch2* expression - *Notch4* expression positively correlates with ER positivity - *Notch1* expression inversely correlates with HER2 expression - Absence of ER expression correlates with higher *Notch3* expression - Notch1 expression inversely correlates with ER and PR expression	- High *Notch2* expression - TNBCs highly express *Notch1–3* - Absence of ER expression correlates with higher *Notch3* expression - Notch1 expression in 100% of TNBCs assessed - Notch4 expression in 73% of TNBCs assessed - Low Notch3 expression in TNBC compared to luminal A tumors - Notch1 enriched in the basal subtype	- High *Notch2* expression - *Notch4* expression positively correlates with ER positivity - *Notch1* expression inversely correlates with HER2 expression - High Notch3 expression compared to TNBC - Notch1 expression inversely correlates with ER and PR expression	- High *Notch2* expression - *Notch4* expression positively correlates with ER positivity - *Notch1* expression inversely correlates with HER2 expression - Notch1 expression inversely correlates with ER and PR expression

*Note*. Adapted from “Moving breast cancer therapy up a notch,” by Mollen EWJ, Ient J, Tjan-Heijnen VCG, Boersma LJ, Miele L, Smidt ML, et al. Front Oncol. 2018;8:518 (https://www.frontiersin.org/articles/10.3389/fonc.2018.00518/full). CC BY.

The Notch signaling pathway is mediated by one of four Notch receptors (Notch1–4). While Notch1 and Notch2 are ubiquitously expressed throughout development and in adult life, Notch3 and Notch4 are more abundant in vasculature cell subtypes. Notch1 and Notch2 knockouts are embryonically lethal due to multiple organ defects, while Notch3 and Notch4 knockouts are viable but display subtle vascular abnormalities [147]. Five Notch ligands are recognized in mammals: delta-like ligands 1 (DLL1), DLL3, DLL4, and Jagged 1 (JAG1) and JAG2 [166].

Depending on the cancer type, the Notch pathway can be hyper- or hypo-activated, which relies on specific Notch receptors. In some organs, both pathway activation and repression have been observed, depending on the tumor subtype or model system in question [168].

Overexpression of Notch1 and/or JAG1 have been associated with poor overall survival, supporting the role of Notch as a prognostic biomarker in BC [167]. While Notch gain-of-function mutations are present in a limited number of BCs, *Notch* is overexpressed, activated, and crosstalks with other oncogenic pathways in several breast tumors [165].

**Figure 7.**
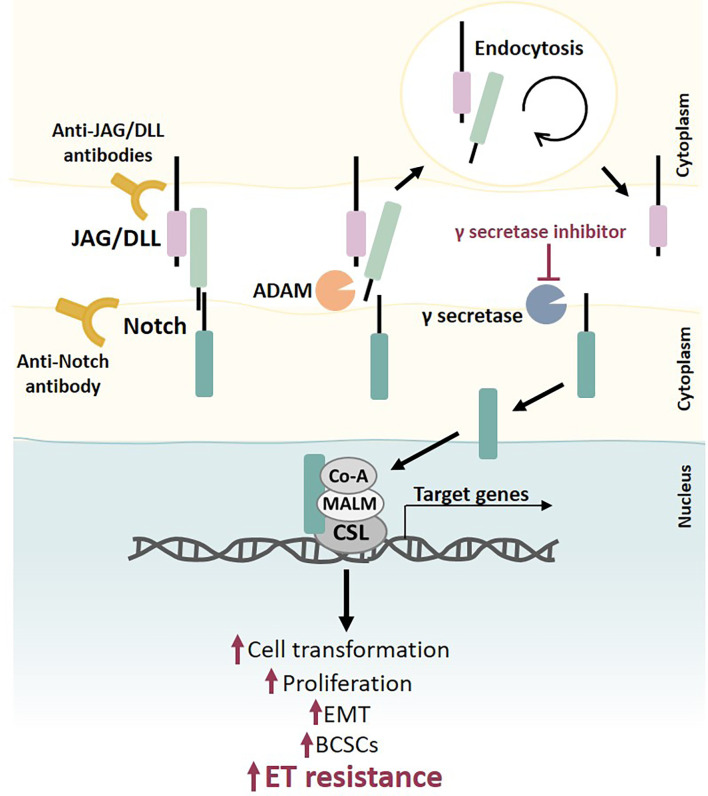
The role of Notch signaling in ET resistance. The Notch receptor is activated by binding to a ligand (JAG/DLL) presented in a neighboring cell. This interaction removes the extracellular portion of Notch from the transmembrane portion, resulting in its endocytosis followed by cleavage events by a disintegrin and metalloprotease (ADAM) and then by γ secretase, which allows the release of the intracellular Notch portion. This intracellular portion translocates to the nucleus, where it binds to DNA-binding protein CSL and recruits transcriptional coactivator Mastermind-like proteins (MALM) and other transcriptional coactivators to initiate transcription of *Notch* target genes. Notch regulates several cellular processes, and dysregulation of this pathway (e.g., through Notch receptor mutations, overexpression of ligands and/or receptors, and/or overexpression of target genes) contributes to increased cell transformation, proliferation, EMT, BCSCs population, and drug resistance, namely ET resistance. Notch signaling inhibition can be accomplished by using different molecules, such as γ-secretase inhibitors and antibodies anti-Notch ligands, and/or receptors. Co-A: coactivator

Some of the earliest known targets of Notch signaling include transcriptional repressors, such as the hairy/enhancer of split (*HES*) genes, the HES subfamily members HEY1, HEY2, and HEYL, c-Myc, and cyclin D1 (Figure 7) [167, 168].

The Notch pathway is involved in breast tumorigenesis, enhanced BC stem cell (BCSC) self-renewal and proliferation, promotion of EMT, angiogenesis, and immunomodulation [168, 169]. However, depending on the BC subtype and/or context, Notch can have an oncogenic or tumor suppressor role [166]. Dysregulation of Notch signaling, namely through activating Notch receptor activating mutations, overexpression of ligands and/or receptors, and/or overexpression of target genes, contributes to increased proliferation, cell transformation, and drug resistance in tumors such as breast, multiple myeloma, prostate, T-cell acute lymphoblastic leukemia, among others [167].

Cumulative evidence indicates that cancer stem cells (CSCs) are key drivers of acquired endocrine resistance in ERα^+^ breast tumors. Endocrine-resistant BC shows an increase in BCSCs with Notch3/4 expression [165].

Estradiol has been shown to inhibit Notch activity by affecting the receptor cellular location, and TAM and raloxifene have been shown to block this effect, thereby activating the Notch pathway. Therefore, the Notch pathway can be hyperactivated in resistant BC cells, and this can be abrogated by blocking this pathway [166]. Taken together, this provides a strong rationale for studies combining Notch inhibitors with current BC treatment modalities [165].

Interaction between cellular components of the TME and BC cells also regulates Notch signaling-driven therapeutic resistance in breast tumors. Different cellular components of the TME can induce CSC survival, stemness, and resistance through either transforming growth factor beta (TGF-β)-dependent mechanisms or by releasing soluble factors such as cytokines, chemokines, and growth factors that favor angiogenesis and an immunosuppressive environment. In turn, all these factors augment Notch ligand- and receptor-mediated chemoresistance, endocrine resistance, and radioresistance in breast tumors. Additionally, cancer-associated fibroblasts and tumor-associated macrophages can collaborate via cell-cell interaction to promote endocrine resistance, with Notch signaling potentially contributing to the crosstalk between these two cell types [166].

## Conclusions

ET was the first targeted treatment developed in BC, and a long and thrilling journey has been traveled since the 1950s when the understanding of ER’s role in disease and its potential as a predictive biomarker began to change the treatment landscape of the disease. However, this remarkable treatment does not provide a cure for all patients, even in association with other types of treatment. Several mechanisms involving ERs, cell cycle and apoptosis regulators, and TKRs have been clearly implicated in endocrine resistance, with numerous drugs and combinations explored to overcome such resistance. Numerous clinical trials have been conducted in this setting, some of which with positive results that led to the approval of first and second line therapies to overcome resistance in BC.

It is clear that there are promising mechanisms and biomarkers of ET resistance that lack clinical validation. Deep analysis of clinical cohorts could expand the portfolio of potential biomarkers, a clear unmet need. However, the clinical and biological heterogeneity of large patient cohorts is challenging in terms of interpretation and clear identification of robust biomarkers. Technologies like next-generation sequencing (NGS) and digital droplet polymerase chain reaction (ddPCR), along with modern bioinformatics pipelines, are key for future research.

Moreover, the TME which includes a panoply of cellular and noncellular (matrix, cytokines, and physical) components affects the response to cancer therapy, namely ET. The communication between cells and the extracellular matrix, along with cell-to-cell communication, is critical for cellular transcriptomics and pathway activity. Therefore, pre-clinical research on the mechanism of resistance must evolve from two-dimensional (2D) cell culture models to heterocellular three-dimensional (3D) models. In this sense, the use of patient-derived organoids also represents an important tool.

Furthermore, the immune component of the TME has particular relevance in response to therapy. In addition, immunotherapy is gaining relevance in the context of ER^+^ BC. Unfortunately, patient-derived xenografts (PDXs), while undoubtedly useful for screening drug response, lack the immune component, unless conducted in humanized mice. The development of humanized mice at affordable costs will certainly accelerate research in this setting.

Overall, the discovery and understanding of these additional resistance mechanisms will predictably lead to even better outcomes for BC patients. Evidence of the role of new putative mediators of ET resistance, like RANKL-RANK, NFκB, and Notch pathways, supports further studies in the area.

## Abbreviations

A/B domain: amino-terminal domain
AIs: aromatase inhibitors
AKT: protein kinase B
AP-1: activator protein 1
BC: breast cancer
BCL-2: B-cell lymphoma 2
BCSC: breast cancer stem cell
CDK4/6i: cyclin-dependent kinase 4/6 inhibitors
CDKs: cyclin-dependent kinases
DBD: DNA binding domain
D-domain: hinge region domain
DLL1: delta-like ligands 1
EGFR: epidermal growth factor receptor
EMT: epithelial-mesenchymal transition
ER^+^: estrogen receptor-positive
ERK: extracellular signal-regulated kinase
ESR1: estrogen receptor 1
ET: endocrine therapy
F-domain: carboxyl-terminal domain
FGFRs: fibroblast growth factor receptors
FULV: fulvestrant
HER2^+^: human epidermal growth factor receptor 2-positive
HR^+^: hormone receptor-positive
IκB: nuclear factor kappa B inhibitor
IKKα: nuclear factor kappa B kinase α
IL-1: interleukin-1
JAG1: Jagged 1
JNK: c-jun N-terminal kinase
LBD: ligand-binding domain
MAPK: mitogen-activated protein kinase
MMTV: mouse mammary tumor virus
mOS: median overall survival
mPFS: median progression-free survival
mRNA: messenger RNA
mTOR: mammalian target of rapamycin
NCoA1: nuclear receptor coactivator 1
NFκB: nuclear factor kappa B
NIK: nuclear factor kappa B-inducing kinase
NR2F2: nuclear receptor subfamily 2 group F member 2
PFS: progression-free survival
PI3K: phosphatidylinositol 3-kinase
PIK3CA: phosphatidylinositol-4,5-bisphosphate 3-kinase catalytic subunit alpha
PR^+^: progesterone receptor-positive
RANK: receptor activator of nuclear factor kappa B
RANK OE: receptor activator of nuclear factor kappa B-overexpressing
RANKL: receptor activator of nuclear factor kappa B ligand
SERDs: selective estrogen receptor downregulators
SERMs: selective estrogen receptor modulators
TAM: tamoxifen
TKRs: tyrosine kinase receptors
TME: tumor microenvironment
TNBCs: triple-negative breast cancers
TNF: tumor necrosis factor

## Declarations

### Copyright

© The Author(s) 2022.

## References

[1] Sung H Ferlay J Siegel RL Laversanne M Soerjomataram I Jemal A Global cancer statistics 2020: GLOBOCAN estimates of incidence and mortality worldwide for 36 cancers in 185 countries. CA Cancer J Clin. 2021;71:209–49. 10.3322/caac.21660 33538338

[2] Harbeck N Gnant M. Breast cancer. Lancet. 2017;389:1134–50. 10.1016/S0140-6736(16)31891-8 27865536

[3] Giuliano M Schifp R Osborne CK Trivedi MV. Biological mechanisms and clinical implications of endocrine resistance in breast cancer. Breast. 2011;20 Suppl 3:S42–9. 10.1016/S0960-9776(11)70293-4 22015292

[4] Rani A Stebbing J Giamas G Murphy J. Endocrine resistance in hormone receptor positive breast cancer—from mechanism to therapy. Front Endocrinol (Lausanne). 2019;10:245. 10.3389/fendo.2019.00245 31178825 PMC6543000

[5] Haque MM Desai KV. Pathways to endocrine therapy resistance in breast cancer. Front Endocrinol (Lausanne). 2019;10:573. 10.3389/fendo.2019.00573 31496995 PMC6712962

[6] Musgrove EA Sutherland RL. Biological determinants of endocrine resistance in breast cancer. Nat Rev Cancer. 2009;9:631–43. 10.1038/nrc2713 19701242

[7] Guillette TC Jackson TW Belcher SM. Duality of estrogen receptor β action in cancer progression. Curr Opin Pharmacol. 2018;41:66–73. 10.1016/j.coph.2018.05.001 29772419 PMC8008732

[8] Speirs V Parkes AT Kerin MJ Walton DS Carleton PJ Fox JN Coexpression of estrogen receptor alpha and beta: poor prognostic factors in human breast cancer? Cancer Res. 1999;59:525–8. 9973193

[9] Speirs V Malone C Walton DS Kerin MJ Atkin SL. Increased expression of estrogen receptor beta mRNA in tamoxifen-resistant breast cancer patients. Cancer Res. 1999;59:5421–4. 10554009

[10] Guo L Zhang Y Zhang W Yilamu D. Correlation between estrogen receptor β expression and the curative effect of endocrine therapy in breast cancer patients. Exp Ther Med. 2014;7:1568–72. 10.3892/etm.2014.1634 24926345 PMC4043576

[11] Guo L Zhang YU Yilamu D Liu S Guo C. ERβ overexpression results in endocrine therapy resistance and poor prognosis in postmenopausal ERα-positive breast cancer patients. Oncol Lett. 2016;11:1531–6. 10.3892/ol.2016.4095 26893775 PMC4734268

[12] Belachew EB Sewasew DT. Molecular mechanisms of endocrine resistance in estrogen-positive breast cancer. Front Endocrinol (Lausanne). 2021;12:599586. Erratum in: Front Endocrinol (Lausanne). 2021;12:689705. 10.3389/fendo.2021.599586 33841325 PMC8030661

[13] Naik N Madani A Esteva A Keskar NS Press MF Ruderman D Deep learning-enabled breast cancer hormonal receptor status determination from base-level H&E stains. Nat Commun. 2020;11:5727. 10.1038/s41467-020-19334-3 33199723 PMC7670411

[14] Ali S Coombes RC. Endocrine-responsive breast cancer and strategies for combating resistance. Nat Rev Cancer. 2002;2:101–12. 10.1038/nrc721 12635173

[15] Zattarin E Leporati R Ligorio F Lobefaro R Vingiani A Pruneri G Hormone receptor loss in breast cancer: molecular mechanisms, clinical settings, and therapeutic implications. Cells. 2020;9:2644. 10.3390/cells9122644 33316954 PMC7764472

[16] Bardou VJ Arpino G Elledge RM Osborne CK Clark GM. Progesterone receptor status significantly improves outcome prediction over estrogen receptor status alone for adjuvant endocrine therapy in two large breast cancer databases. J Clin Oncol. 2003;21:1973–9. 10.1200/JCO.2003.09.099 12743151

[17] Ueno T Saji S Chiba T Kamma H Isaka H Itoh H Progesterone receptor expression in proliferating cancer cells of hormone-receptor-positive breast cancer. Tumour Biol. 2018;40:1010428318811025. 10.1177/1010428318811025 30841783

[18] Kunc M Popęda M Biernat W Senkus E. Lost but not least—novel insights into progesterone receptor loss in estrogen receptor-positive breast cancer. Cancers (Basel). 2021;13:4755. 10.3390/cancers13194755 34638241 PMC8507533

[19] Ma CX Sanchez CG Ellis MJ. Predicting endocrine therapy responsiveness in breast cancer. Oncology (Williston Park). 2009;23:133–42. 19323294

[20] Ring A Dowsett M. Mechanisms of tamoxifen resistance. Endocr Relat Cancer. 2004;11:643–58. 10.1677/erc.1.00776 15613444

[21] Hoskins JM Carey LA McLeod HL. CYP2D6 and tamoxifen: DNA matters in breast cancer. Nat Rev Cancer. 2009;9:576–86. 10.1038/nrc2683 19629072

[22] Riggins RB Schrecengost RS Guerrero MS Bouton AH. Pathways to tamoxifen resistance. Cancer Lett. 2007;256:1–24. 10.1016/j.canlet.2007.03.016 17475399 PMC2533271

[23] Luque-Bolivar A Pérez-Mora E Villegas VE Rondón-Lagos M. Resistance and overcoming resistance in breast cancer. Breast Cancer (Dove Med Press). 2020;12:211–29. 10.2147/BCTT.S270799 33204149 PMC7666993

[24] Mills JN Rutkovsky AC Giordano A. Mechanisms of resistance in estrogen receptor positive breast cancer: overcoming resistance to tamoxifen/aromatase inhibitors. Curr Opin Pharmacol. 2018;41:59–65. 10.1016/j.coph.2018.04.009 29719270 PMC6454890

[25] Clarke R Shajahan AN Riggins RB Cho Y Crawford A Xuan J Gene network signaling in hormone responsiveness modifies apoptosis and autophagy in breast cancer cells. J Steroid Biochem Mol Biol. 2009;114:8–20. 10.1016/j.jsbmb.2008.12.023 19444933 PMC2768542

[26] Zhao M Ramaswamy B. Mechanisms and therapeutic advances in the management of endocrine-resistant breast cancer. World J Clin Oncol. 2014;5:248–62. 10.5306/wjco.v5.i3.248 25114842 PMC4127598

[27] Liu CY Wu CY Petrossian K Huang TT Tseng LM Chen S. Treatment for the endocrine resistant breast cancer: current options and future perspectives. J Steroid Biochem Mol Biol. 2017;172:166–75. 10.1016/j.jsbmb.2017.07.001 28684381

[28] AlFakeeh A Brezden-Masley C. Overcoming endocrine resistance in hormone receptor-positive breast cancer. Curr Oncol. 2018;25:S18–27. 10.3747/co.25.3752 29910644 PMC6001756

[29] Masuda N Nishimura R Takahashi M Inoue K Ohno S Iwata H Palbociclib in combination with letrozole as first-line treatment for advanced breast cancer: a Japanese phase II study. Cancer Sci. 2018;109:803–13. 10.1111/cas.13507 29345736 PMC5834809

[30] Lv Q Guan S Zhu M Huang H Wu J Dai X. FGFR1 is associated with tamoxifen resistance and poor prognosis of ER-positive breast cancers by suppressing ER protein expression. Technol Cancer Res Treat. 2021;20:15330338211004935. 10.1177/15330338211004935 33783288 PMC8013883

[31] Dong C Wu J Chen Y Nie J Chen C. Activation of PI3K/AKT/mTOR pathway causes drug resistance in breast cancer. Front Pharmacol. 2021;12:628690. 10.3389/fphar.2021.628690 33790792 PMC8005514

[32] Herynk MH Fuqua SA. Estrogen receptor mutations in human disease. Endocr Rev 2004;25:869–98. 10.1210/er.2003-0010 15583021

[33] Iwase H Greenman JM Barnes DM Bobrow L Hodgson S Mathew CG. Loss of heterozygosity of the oestrogen receptor gene in breast cancer. Br J Cancer. 1995;71:448–50. 10.1038/bjc.1995.91 7880722 PMC2033633

[34] Adams BD Furneaux H White BA. The micro-ribonucleic acid (miRNA) miR-206 targets the human estrogen receptor-α (ERα) and represses ERα messenger RNA and protein expression in breast cancer cell lines. Mol Endocrinol 2007;21:1132–47. 10.1210/me.2007-0022 17312270

[35] Shi L Dong B Li Z Lu Y Ouyang T Li J Expression of ER-α36, a novel variant of estrogen receptor α, and resistance to tamoxifen treatment in breast cancer. J Clin Oncol. 2009;27:3423–9. DOI: 10.1200/JCO.2008.17.2254 19487384 PMC2717750

[36] Gururaj AE Rayala SK Vadlamudi RK Kumar R. Novel mechanisms of resistance to endocrine therapy: genomic and nongenomic considerations. Clin Cancer Res. 2006;12:1001s–7s. 10.1158/1078-0432.CCR-05-2110 16467116

[37] Lavinsky RM Jepsen K Heinzel T Torchia J Mullen TM Schiff R Diverse signaling pathways modulate nuclear receptor recruitment of N-CoR and SMRT complexes. Proc Natl Acad Sci U S A. 1998;95:2920–5. 10.1073/pnas.95.6.2920 9501191 PMC19670

[38] Shou J Massarweh S Osborne CK Wakeling AE Ali S Weiss H Mechanisms of tamoxifen resistance: increased estrogen receptor-HER2/neu cross-talk in ER/HER2-positive breast cancer. J Natl Cancer Inst. 2004;96:926–35. 10.1093/jnci/djh166 15199112

[39] Osborne CK Bardou V Hopp TA Chamness GC Hilsenbeck SG Fuqua SA Role of the estrogen receptor coactivator AIB1 (SRC-3) and HER-2/neu in tamoxifen resistance in breast cancer. J Natl Cancer Inst. 2003;95:353–61. 10.1093/jnci/95.5.353 12618500

[40] Zhou Y Yau C Gray JW Chew K Dairkee SH Moore DH Enhanced NFκB and AP-1 transcriptional activity associated with antiestrogen resistant breast cancer. BMC Cancer. 2007;7:59. 10.1186/1471-2407-7-59 17407600 PMC1852565

[41] Schiff R Reddy P Ahotupa M Coronado-Heinsohn E Grim M Hilsenbeck SG Oxidative stress and AP-1 activity in tamoxifen-resistant breast tumors *in vivo*. J Natl Cancer Inst. 2000;92:1926–34. 10.1093/jnci/92.23.1926 11106684

[42] Lupien M Meyer CA Bailey ST Eeckhoute J Cook J Westerling T Growth factor stimulation induces a distinct ERα cistrome underlying breast cancer endocrine resistance. Genes Dev. 2010;24:2219–27. 10.1101/gad.1944810 20889718 PMC2947773

[43] Massarweh S Osborne CK Creighton CJ Qin L Tsimelzon A Huang S Tamoxifen resistance in breast tumors is driven by growth factor receptor signaling with repression of classic estrogen receptor genomic function. Cancer Res. 2008;68:826–33. 10.1158/0008-5472.CAN-07-2707 18245484

[44] Arpino G Green SJ Allred DC Lew D Martino S Osborne CK HER-2 amplification, HER-1 expression, and tamoxifen response in estrogen receptor-positive metastatic breast cancer: a southwest oncology group study. Clin Cancer Res. 2004;10:5670–6. 10.1158/1078-0432.CCR-04-0110 15355892

[45] Konecny G Pauletti G Pegram M Untch M Dandekar S Aguilar Z Quantitative association between HER-2/neu and steroid hormone receptors in hormone receptor-positive primary breast cancer. J Natl Cancer Inst. 2003;95:142–53. 10.1093/jnci/95.2.142 12529347

[46] De Laurentiis M Arpino G Massarelli E Ruggiero A Carlomagno C Ciardiello F A meta-analysis on the interaction between HER-2 expression and response to endocrine treatment in advanced breast cancer. Clin Cancer Res. 2005;11:4741–8. 10.1158/1078-0432.CCR-04-2569 16000569

[47] Miller TW Pérez-Torres M Narasanna A Guix M Stål O Pérez-Tenorio G Loss of Phosphatase and Tensin homologue deleted on chromosome 10 engages ErbB3 and insulin-like growth factor-I receptor signaling to promote antiestrogen resistance in breast cancer. Cancer Res. 2009;69:4192–201. 10.1158/0008-5472.CAN-09-0042 19435893 PMC2724871

[48] Cui X Zhang P Deng W Oesterreich S Lu Y Mills GB Insulin-like growth factor-I inhibits progesterone receptor expression in breast cancer cells via the phosphatidylinositol 3-kinase/Akt/mammalian target of rapamycin pathway: progesterone receptor as a potential indicator of growth factor activity in breast cancer. Mol Endocrinol. 2003;17:575–88. 10.1210/me.2002-0318 12554765

[49] Campbell RA Bhat-Nakshatri P Patel NM Constantinidou D Ali S Nakshatri H. Phosphatidylinositol 3-kinase/AKT-mediated activation of estrogen receptor α: a new model for anti-estrogen resistance. J Biol Chem. 2001;276:9817–24. 10.1074/jbc.M010840200 11139588

[50] DeGraffenried LA Friedrichs WE Fulcher L Fernandes G Silva JM Peralba JM Eicosapentaenoic acid restores tamoxifen sensitivity in breast cancer cells with high Akt activity. Ann Oncol. 2003;14:1051–6. 10.1093/annonc/mdg291 12853346

[51] deGraffenried LA Friedrichs WE Russell DH Donzis EJ Middleton AK Silva JM Inhibition of mTOR activity restores tamoxifen response in breast cancer cells with aberrant Akt Activity. Clin Cancer Res. 2004;10:8059–67. 10.1158/1078-0432.CCR-04-0035 15585641

[52] Creighton CJ Fu X Hennessy BT Casa AJ Zhang Y Gonzalez-Angulo AM Proteomic and transcriptomic profiling reveals a link between the PI3K pathway and lower estrogen-receptor (ER) levels and activity in ER^+^ breast cancer. Breast Cancer Res. 2010;12:R40. 10.1186/bcr2594 20569503 PMC2917035

[53] Kato S Endoh H Masuhiro Y Kitamoto T Uchiyama S Sasaki H Activation of the estrogen receptor through phosphorylation by mitogen-activated protein kinase. Science. 1995;270:1491–4. 10.1126/science.270.5241.1491 7491495

[54] Bunone G Briand PA Miksicek RJ Picard D. Activation of the unliganded estrogen receptor by EGF involves the MAP kinase pathway and direct phosphorylation. EMBO J. 1996;15:2174–83. 10.1002/j.1460-2075.1996.tb00571.x 8641283 PMC450141

[55] Gutierrez MC Detre S Johnston S Mohsin SK Shou J Allred DC Molecular changes in tamoxifen-resistant breast cancer: relationship between estrogen receptor, HER-2, and p38 mitogen-activated protein kinase. J Clin Oncol. 2005;23:2469–76. 10.1200/JCO.2005.01.172 15753463

[56] Butt AJ McNeil CM Musgrove EA Sutherland RL. Downstream targets of growth factor and oestrogen signalling and endocrine resistance: the potential roles of c-Myc, cyclin D1 and cyclin E. Endocr Relat Cancer. 2005;12 Suppl 1:S47–59. 10.1677/erc.1.00993 16113099

[57] Chu IM Hengst L Slingerland JM. The Cdk inhibitor p27 in human cancer: prognostic potential and relevance to anticancer therapy. Nat Rev Cancer. 2008;8:253–67. 10.1038/nrc2347 18354415

[58] Pérez-Tenorio G Berglund F Merca AE Nordenskjöld B Rutqvist LE Skoog L Cytoplasmic p21WAF1/CIP1 correlates with Akt activation and poor response to tamoxifen in breast cancer. Int J Oncol. 2006;28:1031–42. 10.3892/ijo.28.5.1031 16596219

[59] Kumar R Mandal M Lipton A Harvey H Thompson CB. Overexpression of HER2 modulates bcl-2, bcl-XL, and tamoxifen-induced apoptosis in human MCF-7 breast cancer cells. Clin Cancer Res. 1996;2:1215–9. 9816290

[60] Hartkopf AD Grischke EM Brucker SY. Endocrine-resistant breast cancer: mechanisms and treatment. Breast Care. 2020;15:347–54. 10.1159/000508675 32982644 PMC7490658

[61] Clarke R Tyson JJ Dixon JM. Endocrine resistance in breast cancer—an overview and update. Mol Cell Endocrinol. 2015;418:220–34. 10.1016/j.mce.2015.09.035 26455641 PMC4684757

[62] Zhang QX Borg A Wolf DM Oesterreich S Fuqua SA. An estrogen receptor mutant with strong hormone-independent activity from a metastatic breast cancer. Cancer Res. 1997;57:1244–9. 9102207

[63] Hanker AB Sudhan DR Arteaga CL. Overcoming endocrine resistance in breast cancer. Cancer Cell. 2020;37:496–513. 10.1016/j.ccell.2020.03.009 32289273 PMC7169993

[64] Jeselsohn R Buchwalter G De Angelis C Brown M Schiff R. ESR1 mutations—a mechanism for acquired endocrine resistance in breast cancer. Nat Rev Clin Oncol. 2015;12:573–83. 10.1038/nrclinonc.2015.117 26122181 PMC4911210

[65] Katzenellenbogen JA Mayne CG Katzenellenbogen BS Greene GL Chandarlapaty S. Structural underpinnings of estrogen receptor mutations in endocrine therapy resistance. Nat Rev Cancer. 2018;18:377–88. Erratum in: Nat Rev Cancer. 2018;18:662. 10.1038/s41568-018-0001-z 29662238 PMC6252060

[66] Hua H Zhang H Kong Q Jiang Y. Mechanisms for estrogen receptor expression in human cancer. Exp Hematol Oncol. 2018;7:24. 10.1186/s40164-018-0116-7 30250760 PMC6148803

[67] Nair BC Vadlamudi RK. Regulation of hormonal therapy resistance by cell cycle machinery. Gene Ther Mol Biol. 2008;12:395. 20148177 PMC2817953

[68] Lange CA Yee D. Killing the second messenger: targeting loss of cell cycle control in endocrine-resistant breast cancer. Endocr Relat Cancer. 2011;18:C19–24. 10.1530/ERC-11-0112 21613412 PMC3924782

[69] Finn RS Martin M Rugo HS Jones S Im SA Gelmon K Palbociclib and letrozole in advanced breast cancer. N Engl J Med. 2016;375:1925–36. 10.1056/NEJMoa1607303 27959613

[70] Tripathy D Im SA Colleoni M Franke F Bardia A Harbeck N Ribociclib plus endocrine therapy for premenopausal women with hormone-receptor-positive, advanced breast cancer (MONALEESA-7): a randomised phase 3 trial. Lancet Oncol. 2018;19:904–15. 10.1016/S1470-2045(18)30292-4 29804902

[71] André F Ciruelos EM Juric D Loibl S Campone M Mayer IA Alpelisib plus fulvestrant for *PIK3CA*-mutated, hormone receptor-positive, human epidermal growth factor receptor-2-negative advanced breast cancer: final overall survival results from SOLAR-1. Ann Oncol. 2021;32:208–17. 10.1016/j.annonc.2020.11.011 33246021

[72] Turner NC Slamon DJ Ro J Bondarenko I Im SA Masuda N Overall survival with palbociclib and fulvestrant in advanced breast cancer. N Engl J Med. 2018;379:1926–36. 10.1056/NEJMoa1810527 30345905

[73] Sledge GW Jr Toi M Neven P Sohn J Inoue K Pivot X MONARCH 2: abemaciclib in combination with fulvestrant in women with HR^+^/HER2^–^ advanced breast cancer who had progressed while receiving endocrine therapy. J Clin Oncol. 2017;35:2875–84. 10.1200/JCO.2017.73.7585 28580882

[74] Cristofanilli M Turner NC Bondarenko I Ro J Im SA Masuda N Fulvestrant plus palbociclib *versus* fulvestrant plus placebo for treatment of hormone-receptor-positive, HER2-negative metastatic breast cancer that progressed on previous endocrine therapy (PALOMA-3): final analysis of the multicentre, double-blind, phase 3 randomised controlled trial. Lancet Oncol. 2016;17:425–39. Erratum in: Lancet Oncol. 2016;17:e136. Erratum in: Lancet Oncol. 2016;17:e270. 10.1016/S1470-2045(15)00613-0 26947331

[75] Slamon DJ Neven P Chia S Fasching PA De Laurentiis M Im SA Phase III randomized study of ribociclib and fulvestrant in hormone receptor-positive, human epidermal growth factor receptor 2-negative advanced breast cancer: MONALEESA-3. J Clin Oncol. 2018;36:2465–72. 10.1200/JCO.2018.78.9909 29860922

[76] Slamon DJ Neven P Chia S Fasching PA De Laurentiis M Im SA Overall survival with ribociclib plus fulvestrant in advanced breast cancer. N Engl J Med. 2020;382:514–24. 10.1056/NEJMoa1911149 31826360

[77] Sledge GW Jr Toi M Neven P Sohn J Inoue K Pivot X The effect of abemaciclib plus fulvestrant on overall survival in hormone receptor-positive, ERBB2-negative breast cancer that progressed on endocrine therapy-MONARCH 2: a randomized clinical trial. JAMA Oncol. 2020;6:116–24. 10.1001/jamaoncol.2019.4782 31563959 PMC6777264

[78] Rugo HS Finn RS Diéras V Ettl J Lipatov O Joy AA Palbociclib plus letrozole as first-line therapy in estrogen receptor-positive/human epidermal growth factor receptor 2-negative advanced breast cancer with extended follow-up. Breast Cancer Res Treat. 2019;174:719–29. 10.1007/s10549-018-05125-4 30632023 PMC6438948

[79] Hortobagyi GN Stemmer SM Burris HA Yap YS Sonke GS Paluch-Shimon S Updated results from MONALEESA-2, a phase III trial of first-line ribociclib plus letrozole versus placebo plus letrozole in hormone receptor-positive, HER2-negative advanced breast cancer. Ann Oncol. 2018;29:1541–7. Erratum in: Ann Oncol. 2019;30:1842. 10.1093/annonc/mdy155 29718092

[80] Im SA Lu YS Bardia A Harbeck N Colleoni M Franke F Overall survival with ribociclib plus endocrine therapy in breast cancer. N Engl J Med. 2019;381:307–16. 10.1056/NEJMoa1903765 31166679

[81] Johnston S O’Shaughnessy J Martin M Huober J Toi M Sohn J Abemaciclib as initial therapy for advanced breast cancer: MONARCH 3 updated results in prognostic subgroups. NPJ Breast Cancer. 2021;7:80. 10.1038/s41523-021-00289-7 34158513 PMC8219718

[82] Baselga J Campone M Piccart M Burris HA 3rd Rugo HS Sahmoud T Everolimus in postmenopausal hormone-receptor-positive advanced breast cancer. N Engl J Med. 2012;366:520–9. 10.1056/NEJMoa1109653 22149876 PMC5705195

[83] Schmid P Zaiss M Harper-Wynne C Ferreira M Dubey S Chan S Fulvestrant plus vistusertib *vs* fulvestrant plus everolimus *vs* fulvestrant alone for women with hormone receptor-positive metastatic breast cancer: the MANTA phase 2 randomized clinical trial. JAMA Oncol. 2019;5:1556–64. Erratum in: JAMA Oncol. 2021;7:312. 10.1001/jamaoncol.2019.2526 31465093 PMC6865233

[84] Kornblum N Zhao F Manola J Klein P Ramaswamy B Brufsky A Randomized phase II trial of fulvestrant plus everolimus or placebo in postmenopausal women with hormone receptor-positive, human epidermal growth factor receptor 2-negative metastatic breast cancer resistant to aromatase inhibitor therapy: results of PrE0102. J Clin Oncol. 2018;36:1556–63. 10.1200/JCO.2017.76.9331 29664714 PMC7186582

[85] André F Ciruelos E Rubovszky G Campone M Loibl S Rugo HS et al.; SOLAR-1 Study Group. Alpelisib for *PIK3CA*-mutated, hormone receptor-positive advanced breast cancer. N Engl J Med. 2019;380:1929–40. 10.1056/NEJMoa1813904 31091374

[86] Di Leo A Jerusalem G Petruzelka L Torres R Bondarenko IN Khasanov R Results of the CONFIRM phase III trial comparing fulvestrant 250 mg with fulvestrant 500 mg in postmenopausal women with estrogen receptor-positive advanced breast cancer. J Clin Oncol. 2010;28:4594–600. Erratum in: J Clin Oncol. 2011;29:2293. 10.1200/JCO.2010.28.8415 20855825

[87] Chow LWC Lie EF Toi M. Advances in EGFR/HER2-directed clinical research on breast cancer. Adv Cancer Res. 2020;147:375–428. 10.1016/bs.acr.2020.04.009 32593406

[88] Croessmann S Formisano L Kinch LN Gonzalez-Ericsson PI Sudhan DR Nagy RJ Combined blockade of activating *ERBB2* mutations and ER results in synthetic lethality of ER^+^/HER2 mutant breast cancer. Clin Cancer Res. 2019;25:277–89. 10.1158/1078-0432.CCR-18-1544 30314968 PMC6320312

[89] Nayar U Cohen O Kapstad C Cuoco MS Waks AG Wander SA Acquired HER2 mutations in ER^+^ metastatic breast cancer confer resistance to estrogen receptor-directed therapies. Nat Genet. 2019;51:207–16. 10.1038/s41588-018-0287-5 30531871

[90] Razavi P Chang MT Xu G Bandlamudi C Ross DS Vasan N The genomic landscape of endocrine-resistant advanced breast cancers. Cancer Cell. 2018;34:427–38.e6. 10.1016/j.ccell.2018.08.008 30205045 PMC6327853

[91] Smyth LM Piha-Paul SA Won HH Schram AM Saura C Loi S Efficacy and determinants of response to HER kinase inhibition in *HER2*-mutant metastatic breast cancer. Cancer Discov. 2020;10:198–213. 10.1158/2159-8290.CD-19-0966 31806627 PMC7007377

[92] Perez-Garcia J Muñoz-Couselo E Soberino J Racca F Cortes J. Targeting FGFR pathway in breast cancer. Breast. 2018;37:126–33. 10.1016/j.breast.2017.10.014 29156384

[93] Giltnane JM Hutchinson KE Stricker TP Formisano L Young CD Estrada MV Genomic profiling of ER^+^ breast cancers after short-term estrogen suppression reveals alterations associated with endocrine resistance. Sci Transl Med. 2017;9:eaai7993. Erratum in: Sci Transl Med. 2019;11:eaaw7620. 10.1126/scitranslmed.aai7993 28794284 PMC5723145

[94] Chew NJ Lim Kam Sian TCC Nguyen EV Shin SY Yang J Hui MN Evaluation of FGFR targeting in breast cancer through interrogation of patient-derived models. Breast Cancer Res. 2021;23:82. 10.1186/s13058-021-01461-4 34344433 PMC8336364

[95] Turczyk L Kitowska K Mieszkowska M Mieczkowski K Czaplinska D Piasecka D FGFR2-driven signaling counteracts tamoxifen effect on ERα-positive breast cancer cells. Neoplasia. 2017;19:791–804. 10.1016/j.neo.2017.07.006 28869838 PMC5964976

[96] Levine KM Priedigkeit N Basudan A Tasdemir N Sikora MJ Sokol ES FGFR4 overexpression and hotspot mutations in metastatic ER^+^ breast cancer are enriched in the lobular subtype. NPJ Breast Cancer. 2019;5:19. 10.1038/s41523-019-0114-x 31263748 PMC6597581

[97] Garcia-Recio S Thennavan A East MP Parker JS Cejalvo JM Garay JP FGFR4 regulates tumor subtype differentiation in luminal breast cancer and metastatic disease. J Clin Invest. 2020;130:4871–87. 10.1172/JCI130323 32573490 PMC7456247

[98] Alzahrani AS. PI3K/Akt/mTOR inhibitors in cancer: at the bench and bedside. Semin Cancer Biol. 2019;59:125–32. 10.1016/j.semcancer.2019.07.009 31323288

[99] Miller TW Hennessy BT González-Angulo AM Fox EM Mills GB Chen H Hyperactivation of phosphatidylinositol-3 kinase promotes escape from hormone dependence in estrogen receptor-positive human breast cancer. J Clin Invest. 2010;120:2406–13. 10.1172/JCI41680 20530877 PMC2898598

[100] Vitale SR Martorana F Stella S Motta G Inzerilli N Massimino M PI3K inhibition in breast cancer: identifying and overcoming different flavors of resistance. Crit Rev Oncol Hematol. 2021;162:103334. 10.1016/j.critrevonc.2021.103334 33865994

[101] Jones RH Casbard A Carucci M Cox C Butler R Alchami F Fulvestrant plus capivasertib *versus* placebo after relapse or progression on an aromatase inhibitor in metastatic, oestrogen receptor-positive breast cancer (FAKTION): a multicentre, randomised, controlled, phase 2 trial. Lancet Oncol. 2020;21:345–57. 10.1016/S1470-2045(19)30817-4 32035020 PMC7052734

[102] Hyman DM Smyth LM Donoghue MTA Westin SN Bedard PL Dean EJ AKT inhibition in solid tumors with AKT1 mutations. J Clin Oncol. 2017;35:2251–9. Erratum in: J Clin Oncol. 2019;37:360. 10.1200/JCO.2017.73.0143 28489509 PMC5501365

[103] Smyth LM Tamura K Oliveira M Ciruelos EM Mayer IA Sablin MP Capivasertib, an AKT kinase inhibitor, as monotherapy or in combination with fulvestrant in patients with *AKT1*^E17K^-mutant, ER-positive metastatic breast cancer. Clin Cancer Res. 2020;26:3947–57. 10.1158/1078-0432.CCR-19-3953 32312891 PMC7415507

[104] Alves CL Ehmsen S Terp MG Portman N Tuttolomondo M Gammelgaard OL Co-targeting CDK4/6 and AKT with endocrine therapy prevents progression in CDK4/6 inhibitor and endocrine therapy-resistant breast cancer. Nat Commun. 2021;12:5112. Erratum in: Nat Commun. 2021;12:5588. 10.1038/s41467-021-25422-9 34433817 PMC8387387

[105] Hortobagyi GN Chen D Piccart M Rugo HS Burris HA 3rd Pritchard KI Correlative analysis of genetic alterations and everolimus benefit in hormone receptor-positive, human epidermal growth factor receptor 2-negative advanced breast cancer: results from BOLERO-2. J Clin Oncol. 2016;34:419–26. Erratum in: J Clin Oncol. 2019;37:354. Erratum in: J Clin Oncol. 2019;37:355. 10.1200/JCO.2014.60.1971 26503204 PMC5070556

[106] Dougall WC Glaccum M Charrier K Rohrbach K Brasel K De Smedt T RANK is essential for osteoclast and lymph node development. 1999;13:2412–24. 10.1101/gad.13.18.2412 10500098 PMC317030

[107] Nakagawa N Kinosaki M Yamaguchi K Shima N Yasuda H Yano K RANK is the essential signaling receptor for osteoclast differentiation factor in osteoclastogenesis. Biochem Biophys Res Commun. 1998;253:395–400. 10.1006/bbrc.1998.9788 9878548

[108] Lacey DL Boyle WJ Simonet WS Kostenuik PJ Dougall WC Sullivan JK Bench to bedside: elucidation of the OPG-RANK-RANKL pathway and the development of denosumab. Nat Rev Drug Discov. 2012;11:401–19. 10.1038/nrd3705 22543469

[109] Lipton A Goessl C. Clinical development of anti-RANKL therapies for treatment and prevention of bone metastasis. Bone. 2011;48:96–9. 10.1016/j.bone.2010.10.161 20950721

[110] Armstrong AP Miller RE Jones JC Zhang J Keller ET Dougall WC. RANKL acts directly on RANK-expressing prostate tumor cells and mediates migration and expression of tumor metastasis genes. Prostate. 2008;68:92–104. 10.1002/pros.20678 18008334

[111] Casimiro S Mohammad KS Pires R Tato-Costa J Alho I Teixeira R RANKL/RANK/MMP-1 molecular triad contributes to the metastatic phenotype of breast and prostate cancer cells *in vitro*. PLoS One. 2013;8:e63153. 10.1371/journal.pone.0063153 23696795 PMC3656033

[112] Jones DH Nakashima T Sanchez OH Kozieradzki I Komarova SV Sarosi I Regulation of cancer cell migration and bone metastasis by RANKL. Nature. 2006;440:692–6. 10.1038/nature04524 16572175

[113] Mori K Le Goff B Charrier C Battaglia S Heymann D Rédini F. DU145 human prostate cancer cells express functional receptor activator of NFkappaB: new insights in the prostate cancer bone metastasis process. Bone. 2007;40:981–90. 10.1016/j.bone.2006.11.006 17196895

[114] Tanos T Sflomos G Echeverria PC Ayyanan A Gutierrez M Delaloye JF Progesterone/RANKL is a major regulatory axis in the human breast. Sci Transl Med. 2013;5:182ra55. 10.1126/scitranslmed.3005654 23616122

[115] Gonzalez-Suarez E Jacob AP Jones J Miller R Roudier-Meyer MP Erwert R RANK ligand mediates progestin-induced mammary epithelial proliferation and carcinogenesis. Nature. 2010;468:103–7. 10.1038/nature09495 20881963

[116] Schramek D Leibbrandt A Sigl V Kenner L Pospisilik JA Lee HJ Osteoclast differentiation factor RANKL controls development of progestin-driven mammary cancer. Nature. 2010;468:98–102. 10.1038/nature09387 20881962 PMC3084017

[117] Casimiro S Vilhais G Gomes I Costa L. The roadmap of RANKL/RANK pathway in cancer. Cells. 2021;10:1978. 10.3390/cells10081978 34440747 PMC8393235

[118] Pfitzner BM Branstetter D Loibl S Denkert C Lederer B Schmitt WD RANK expression as a prognostic and predictive marker in breast cancer. Breast Cancer Res Treat. 2014;145:307–15. 10.1007/s10549-014-2955-1 24737168

[119] Santini D Perrone G Roato I Godio L Pantano F Grasso D Expression pattern of receptor activator of NFκB (RANK) in a series of primary solid tumors and related bone metastases. J Cell Physiol. 2011;226:780–4. 10.1002/jcp.22402 20857484

[120] Santini D Schiavon G Vincenzi B Gaeta L Pantano F Russo A Receptor activator of NF-kB (RANK) expression in primary tumors associates with bone metastasis occurrence in breast cancer patients. PLoS One. 2011;6:e19234. 10.1371/journal.pone.0019234 21559440 PMC3084800

[121] Zhang L Teng Y Zhang Y Liu J Xu L Qu J Receptor activator for nuclear factor κ B expression predicts poor prognosis in breast cancer patients with bone metastasis but not in patients with visceral metastasis. J Clin Pathol. 2012;65:36–40. 10.1136/jclinpath-2011-200312 22049226

[122] Owen S Ye L Sanders AJ Mason MD Jiang WG. Expression profile of receptor activator of nuclear-κB (RANK), RANK ligand (RANKL) and osteoprotegerin (OPG) in breast cancer. Anticancer Res. 2013;33:199–206. 23267146

[123] Park HS Lee A Chae BJ Bae JS Song BJ Jung SS. Expression of receptor activator of nuclear factor kappa-B as a poor prognostic marker in breast cancer. J Surg Oncol. 2014;110:807–12. 10.1002/jso.23737 25111682

[124] Gomes I de Almeida BP Dâmaso S Mansinho A Correia I Henriques S Expression of receptor activator of NFkB (RANK) drives stemness and resistance to therapy in ER^+^HER2^–^ breast cancer. Oncotarget. 2020;11:1714–28. 10.18632/oncotarget.27576 32477461 PMC7233807

[125] Benítez S Cordero A Santamaría PG Redondo-Pedraza J Rocha AS Collado-Solé A RANK links senescence to stemness in the mammary epithelia, delaying tumor onset but increasing tumor aggressiveness. Dev Cell. 2021;56:1727–41.e7. 10.1016/j.devcel.2021.04.022 34004159 PMC8221814

[126] Pellegrini P Cordero A Gallego MI Dougall WC Muñoz P Pujana MA Constitutive activation of RANK disrupts mammary cell fate leading to tumorigenesis. Stem Cells. 2013;31:1954–65. Erratum in: Stem Cells. 2014;32:1057. Erratum in: Stem Cells. 2014;32:600. 10.1002/stem.1454 23766243

[127] International Prognostic Factors Study Group Lorch A Beyer J Bascoul-Mollevi C Kramar A Einhorn LH Necchi A Prognostic factors in patients with metastatic germ cell tumors who experienced treatment failure with cisplatin-based first-line chemotherapy. J Clin Oncol. 2010;28:4906–11. 10.1200/JCO.2009.26.8128 20956623

[128] Yoldi G Pellegrini P Trinidad EM Cordero A Gomez-Miragaya J Serra-Musach J RANK signaling blockade reduces breast cancer recurrence by inducing tumor cell differentiation. Cancer Res. 2016;76:5857–69. 10.1158/0008-5472.CAN-15-2745 27480274

[129] Cao Y Bonizzi G Seagroves TN Greten FR Johnson R Schmidt EV IKKalpha provides an essential link between RANK signaling and cyclin D1 expression during mammary gland development. Cell. 2001;107:763–75. 10.1016/S0092-8674(01)00599-2 11747812

[130] Sun SC. The noncanonical NF-κB pathway. Immunol Rev. 2012;246:125–40. 10.1111/j.1600-065X.2011.01088.x 22435551 PMC3313452

[131] Sanz-Moreno A Palomeras S Pedersen K Morancho B Pascual T Galván P RANK signaling increases after anti-HER2 therapy contributing to the emergence of resistance in HER2-positive breast cancer. Breast Cancer Res. 2021;23:42. 10.1186/s13058-021-01390-2 33785053 PMC8008631

[132] Tsubaki M Komai M Fujimoto SI Itoh T Imano M Sakamoto K Activation of NF-κB by the RANKL/RANK system up-regulates snail and twist expressions and induces epithelial-to-mesenchymal transition in mammary tumor cell lines. J Exp Clin Cancer Res. 2013;32:62. 10.1186/1756-9966-32-62 24011086 PMC3847095

[133] Pahl HL. Activators and target genes of Rel/NF-kappaB transcription factors. Oncogene. 1999;18:6853–66. 10.1038/sj.onc.1203239 10602461

[134] Zhou Y Eppenberger-Castori S Eppenberger U Benz CC. The NFkappaB pathway and endocrine-resistant breast cancer. Endocr Relat Cancer. 2005;12 Suppl 1:S37–46. 10.1677/erc.1.00977 16113098

[135] Rhodes LV Bratton MR Zhu Y Tilghman SL Muir SE Salvo VA Effects of SDF-1-CXCR4 signaling on microRNA expression and tumorigenesis in estrogen receptor-alpha (ER-α)-positive breast cancer cells. Exp Cell Res. 2011;317:2573–81. 10.1016/j.yexcr.2011.08.016 21906588 PMC3334320

[136] van Agthoven T Veldscholte J Smid M van Agthoven TLA Vreede L Broertjes M Functional identification of genes causing estrogen independence of human breast cancer cells. Breast Cancer Res Treat. 2009;114:23–30. 10.1007/s10549-008-9969-5 18351453

[137] Cao Y Karin M. NF-kappaB in mammary gland development and breast cancer. J Mammary Gland Biol Neoplasia. 2003;8:215–23. 10.1023/A:1025905008934 14635796

[138] Perkins ND. The diverse and complex roles of NF-κB subunits in cancer. Nat Rev Cancer. 2012;12:121–32. 10.1038/nrc3204 22257950

[139] Frasor J Weaver A Pradhan M Dai Y Miller LD Lin CY Positive cross-talk between estrogen receptor and NF-kappaB in breast cancer. Cancer Res 2009;69:8918–25. Erratum in: Cancer Res. 2010;70:854. Erratum in: Cancer Res. 2010;70:2140. 10.1158/0008-5472.CAN-09-2608 19920189 PMC2996265

[140] Nakshatri H Bhat-Nakshatri P Martin DA Goulet RJ Jr Sledge GW Jr. Constitutive activation of NF-kappaB during progression of breast cancer to hormone-independent growth. Mol Cell Biol. 1997;17:3629–39. 10.1128/MCB.17.7.3629 9199297 PMC232215

[141] Nakshatri N Goulet RJ Jr. NF-kappaB and breast cancer. Curr Probl Cancer. 2002;26:282–309. 10.1067/mcn.2002.129977 12429950

[142] Wang CY Guttridge DC Mayo MW Baldwin AS Jr. NF-kappaB induces expression of the Bcl-2 homologue A1/Bfl-1 to preferentially suppress chemotherapy-induced apoptosis. Mol Cell Biol. 1999;19:5923–9. 10.1128/MCB.19.9.5923 10454539 PMC84448

[143] Zhang Q Lenardo MJ Baltimore D. 30 years of NF-κB: a blossoming of relevance to human pathobiology. Cell. 2017;168:37–57. 10.1016/j.cell.2016.12.012 28086098 PMC5268070

[144] Sas L Lardon F Vermeulen PB Hauspy J Van Dam P Pauwels P The interaction between ER and NFκB in resistance to endocrine therapy. Breast Cancer Res. 2012;14:212. 10.1186/bcr3196 22963717 PMC3680926

[145] Khongthong P Roseweir AK Edwards J. The NF-KB pathway and endocrine therapy resistance in breast cancer. Endocr Relat Cancer. 2019;26:R369–80. 10.1530/ERC-19-0087 32013374

[146] Karin M Ben-Neriah Y. Phosphorylation meets ubiquitination: the control of NF-[kappa]B activity. Annu Rev Immunol. 2000;18:621–63. 10.1146/annurev.immunol.18.1.621 10837071

[147] Hoesel B Schmid JA. The complexity of NF-κB signaling in inflammation and cancer. Mol Cancer. 2013;12:86. 10.1186/1476-4598-12-86 23915189 PMC3750319

[148] Wang X Fang Y Sun W Xu Z Zhang Y Wei X Endocrinotherapy resistance of prostate and breast cancer: importance of the NF-κB pathway (review). Int J Oncol. 2020;56:1064–74. 10.3892/ijo.2020.4990 32319568

[149] Pasparakis M Luedde T Schmidt-Supprian M. Dissection of the NF-kappaB signalling cascade in transgenic and knockout mice. Cell Death Differ. 2006;13:861–72. 10.1038/sj.cdd.4401870 16470223

[150] Hayden MS West AP Ghosh S. NF-kappaB and the immune response. Oncogene. 2006;25:6758–80. 10.1038/sj.onc.1209943 17072327

[151] Sun SC. The non-canonical NF-κB pathway in immunity and inflammation. Nat Rev Immunol. 2017;17:545–58. 10.1038/nri.2017.52 28580957 PMC5753586

[152] Maubach G Feige MH Lim MCC Naumann M. NF-kappaB-inducing kinase in cancer. Biochim Biophys Acta Rev Cancer. 2019;1871:40–9. 10.1016/j.bbcan.2018.10.002 30419317

[153] Gray CM Remouchamps C McCorkell KA Solt LA Dejardin E Orange JS Noncanonical NF-κB signaling is limited by classical NF-κB activity. Sci Signal. 2014;7:ra13. 10.1126/scisignal.2004557 24497610 PMC3960999

[154] Yde CW Emdal KB Guerra B Lykkesfeldt AE. NFκB signaling is important for growth of antiestrogen resistant breast cancer cells. Breast Cancer Res Treat. 2012;135:67–78. 10.1007/s10549-012-2053-1 22527100

[155] Rwigemera A Mamelona J Martin LJ. Inhibitory effects of fucoxanthinol on the viability of human breast cancer cell lines MCF-7 and MDA-MB-231 are correlated with modulation of the NF-kappaB pathway. Cell Biol Toxicol. 2014;30:157–67. 10.1007/s10565-014-9277-2 24760606

[156] Helbig G Christopherson KW 2nd Bhat-Nakshatri P Kumar S Kishimoto H Miller KD NF-kappaB promotes breast cancer cell migration and metastasis by inducing the expression of the chemokine receptor CXCR4. J Biol Chem. 2003;278:21631–8. 10.1074/jbc.M300609200 12690099

[157] Pratt MAC Bishop TE White D Yasvinski G Ménard M Niu MY Estrogen withdrawal-induced NF-kappaB activity and bcl-3 expression in breast cancer cells: roles in growth and hormone independence. Mol Cell Biol. 2003;23:6887–900. 10.1128/MCB.23.19.6887-6900.2003 12972607 PMC193930

[158] Fan W Chang J Fu P. Endocrine therapy resistance in breast cancer: current status, possible mechanisms and overcoming strategies. Future Med Chem. 2015;7:1511–9. 10.4155/fmc.15.93 26306654 PMC5558537

[159] Jiang X Shapiro DJ. The immune system and inflammation in breast cancer. Mol Cell Endocrinol. 2014;382:673–82. 10.1016/j.mce.2013.06.003 23791814 PMC4919022

[160] Jiang X. Harnessing the immune system for the treatment of breast cancer. J Zhejiang Univ Sci B. 2014;15:1–15. 10.1631/jzus.B1300264 24390741 PMC3891115

[161] Oida K Matsuda A Jung K Xia Y Jang H Amagai Y Nuclear factor-κB plays a critical role in both intrinsic and acquired resistance against endocrine therapy in human breast cancer cells. Sci Rep. 2014;4:4057. 10.1038/srep04057 24531845 PMC3925966

[162] Nehra R Riggins RB Shajahan AN Zwart A Crawford AC Clarke R. BCL2 and CASP8 regulation by NF-kappaB differentially affect mitochondrial function and cell fate in antiestrogen-sensitive and -resistant breast cancer cells. FASEB J. 2010;24:2040–55. 10.1096/fj.09-138305 20154269 PMC2874480

[163] Cogswell PC Guttridge DC Funkhouser WK Baldwin AS Jr. Selective activation of NF-kappa B subunits in human breast cancer: potential roles for NF-kappa B2/p52 and for Bcl-3. Oncogene. 2000;19:1123–31. 10.1038/sj.onc.1203412 10713699

[164] Prescott JA Cook SJ. Targeting IKKβ in cancer: challenges and opportunities for the therapeutic utilisation of IKKβ inhibitors. Cells. 2018;7:115. 10.3390/cells7090115 30142927 PMC6162708

[165] Mollen EWJ Ient J Tjan-Heijnen VCG Boersma LJ Miele L Smidt ML Moving breast cancer therapy up a notch. Front Oncol. 2018;8:518. 10.3389/fonc.2018.00518 30515368 PMC6256059

[166] Nandi A Chakrabarti R. The many facets of Notch signaling in breast cancer: toward overcoming therapeutic resistance. Genes Dev. 2020;34:1422–38. 10.1101/gad.342287.120 33872192 PMC7608750

[167] BeLow M Osipo C. Notch signaling in breast cancer: a role in drug resistance. Cells. 2020;9:2204. 10.3390/cells9102204 33003540 PMC7601482

[168] Meisel CT Porcheri C Mitsiadis TA. Cancer stem cells, *quo vadis*? The Notch signaling pathway in tumor initiation and progression. Cells. 2020;9:1879. 10.3390/cells9081879 32796631 PMC7463613

[169] Moore G Annett S McClements L Robson T. Top Notch targeting strategies in cancer: a detailed overview of recent insights and current perspectives. Cells. 2020;9:1503. 10.3390/cells9061503 32575680 PMC7349363

